# Oxygen induces the expression of invasion and stress response genes in the anaerobic salmon parasite *Spironucleus salmonicida*

**DOI:** 10.1186/s12915-019-0634-8

**Published:** 2019-03-01

**Authors:** Courtney W. Stairs, Anna Kokla, Ásgeir Ástvaldsson, Jon Jerlström-Hultqvist, Staffan Svärd, Thijs J. G. Ettema

**Affiliations:** 1 Department of Cell and Molecular Biology, Uppsala University, Uppsala, Sweden; 2 Present Address: Department of Plant Biology, Swedish University of Agricultural Sciences (SLU), Almas Allé 5, BioCentrum, room D-444, Uppsala, Sweden; 3 Present Address: Department of Biochemistry and Molecular Biology, Dalhousie University, Halifax, Canada; 4 Laboratory of Microbiology, Department of Agrotechnology and Food Sciences, Wageningen University, Stippeneng 4, 6708WE Wageningen, The Netherlands

**Keywords:** *Spironucleus*, Diplomonads, *Giardia*, Oxygen stress, RNAseq, Anaerobiosis, Protist, Parasitology, Spironucleosis, Lateral gene transfer

## Abstract

**Background:**

*Spironucleus salmonicida* is an anaerobic parasite that can cause systemic infections in Atlantic salmon. Unlike other diplomonad parasites, such as the human pathogen *Giardia intestinalis*, *Spironucleus* species can infiltrate the blood stream of their hosts eventually colonizing organs, skin and gills. How this presumed anaerobe can persist and invade oxygenated tissues, despite having a strictly anaerobic metabolism, remains elusive.

**Results:**

To investigate how *S. salmonicida* response to oxygen stress, we performed RNAseq transcriptomic analyses of cells grown in the presence of oxygen or antioxidant-free medium. We found that over 20% of the transcriptome is differentially regulated in oxygen (1705 genes) and antioxidant-depleted (2280 genes) conditions. These differentially regulated transcripts encode proteins related to anaerobic metabolism, cysteine and Fe-S cluster biosynthesis, as well as a large number of proteins of unknown function. *S. salmonicida* does not encode genes involved in the classical elements of oxygen metabolism (e.g., catalases, superoxide dismutase, glutathione biosynthesis, oxidative phosphorylation). Instead, we found that genes encoding bacterial-like oxidoreductases were upregulated in response to oxygen stress. Phylogenetic analysis revealed some of these oxygen-responsive genes (e.g., *nadh oxidase*, *rubrerythrin*, *superoxide reductase*) are rare in eukaryotes and likely derived from lateral gene transfer (LGT) events into diplomonads from prokaryotes. Unexpectedly, we observed that many host evasion- and invasion-related genes were also upregulated under oxidative stress suggesting that oxygen might be an important signal for pathogenesis.

**Conclusion:**

While oxygen is toxic for related organisms, such as *G. intestinalis*, we find that oxygen is likely a gene induction signal for host invasion- and evasion-related pathways in *S. salmonicida*. These data provide the first molecular evidence for how *S. salmonicida* could tolerate oxic host environments and demonstrate how LGT can have a profound impact on the biology of anaerobic parasites.

**Electronic supplementary material:**

The online version of this article (10.1186/s12915-019-0634-8) contains supplementary material, which is available to authorized users.

## Background

The diplomonads are a diverse assemblage of parasitic and free-living eukaryotes that occupy low-oxygen environments. The best-studied diplomonad is the human parasite *Giardia instestinalis* which annually infects 280 million people worldwide [1]. This non-invasive, microaerophilic parasite colonizes the upper small intestine and causes the disease, giardiasis, which manifests in humans as an acute diarrhea that can develop to a chronic stage [2]. The life cycle comprises two main stages: the trophozoite, the actively replicating symptomatic stage, and the cyst, the environmentally resistant infective stage shed in host stools [2, 3]. Another lineage of parasitic diplomonads includes *Spironucleus* species known to infect a wide array of animals including birds, primates, and mice [4–6]. Some *Spironucleus* species infect piscine species where they cause numerous pathologies (e.g., skin lesions, anorexia, lethargy, abnormal swimming behaviors, and reclusiveness) detrimental to healthy fish rearing and are thus a growing concern for global aquaculture and ornamental fish industries [7–10]. The “salmon killer” *Spironucleus salmonicida* is known to infect the digestive tract of farmed Atlantic salmon, Arctic charr, and Chinook salmon (reviewed in [10]). *Spironucleus* outbreaks in farmed Atlantic salmon populations cause significant damage to the aquaculture economy, and the only treatment for spironucleosis, metronidazole, was banned in Europe in the late 1990s [11] due to its carcinogenic activity [12]. Therefore, advancing our current understanding of this parasite’s biology is essential for developing alternative treatment strategies and to block transmission cycles.

One feature that sets *Spironucleus* species apart from *G. intestinalis*, and most other intestinal protozoan parasites, is its ability to cause systemic infections. While the particular lifecycle is unknown for *S. salmonicida*, some details can be predicted from observations of close relatives. Cysts have been described in some *Spironucleus* species [5, 13]; however, no formal molecular characterizations have been reported. Therefore, the trigger and site of encystation is unknown in piscine *Spironucleus* species. Encystation might occur in the gut and exit the host via the feces favoring a fecal-oral transmission model. Alternatively, encystation could occur on external lesions and is thus transferred between fish in close contact, favoring a skin-oral or skin-gill transmission model. Trophozoites have been observed in the gut, feces, skin, organs, and gills of fish infected with *Spironucleus* species and are the primary life stage observed under laboratory conditions [10, 14]. Once inside the host, the trophozoites are predicted to asexually reproduce in the gut and, in some cases, invade the host’s mucosa to enter the blood stream leading to systemic infections.

The ability of *S. salmonicida* to invade and persist in oxygenated tissues following gut colonization is particularly interesting given that it is classically viewed as an anaerobe. Indeed, molecular studies of *S. salmonicida* revealed a suite of oxygen-sensitive enzymes typically associated with anaerobiosis, some of which function in the mitochondrion-related organelles of *S. salmonicida* [15]. These “hydrogenosomes” have completely lost oxidative phosphorylation and instead rely exclusively on substrate-level phosphorylation to generate ATP [15]. Glucose- or amino acid-derived pyruvate is oxidized to acetyl-CoA via Pyruvate: ferredoxin oxidoreductase (PFO), and electrons are transferred via a Ferredoxin (FER) and [FeFe]-hydrogenase (HYD) ultimately reducing protons to hydrogen. The acetyl-CoA generated in this reaction can be further processed to acetate yielding one molecule of ATP via acetyl-CoA synthetase (ACS). Three accessory maturase proteins (HYD E,F,G) also function in the MRO to promote proper assembly of the Fe-S cluster of HYD. A similar pathway functions in the cytoplasm of *S. salmonicida*; however, the electron carrier between PFO and HYD and also the proteins necessary in HYD maturation are currently unknown. In *G. intestinalis*, these pathways are exclusively localized to the cytoplasm.

While in the blood and tissues of its host, *S. salmonicida* is exposed to higher oxygen levels than those in the gut. Yet, the parasite does not encode proteins related to traditional oxygen defense pathways (e.g., superoxide dismutase, catalase, glutathione metabolism) [16, 17]. It is currently unclear how the organism adapts and thrives in oxygenated tissues throughout its life cycle. To investigate how this parasite tolerates oxidative stress, we performed transcriptional profiling of cultured *S. salmonicida* exposed to oxygen or anti-oxidant depletion. We observed that a large portion of the transcriptome is in fact upregulated in response to oxygen and anti-oxidant deprivation suggesting that this parasite employs a variety of strategies (e.g., oxygen clearance, iron-sequestration, and cysteine metabolism) to thrive in different oxygen tensions. Importantly, we show that some of the genes responsive to oxidative stress were transferred to *S. salmonicida* from bacterial donors via lateral gene transfer (LGT), thereby showcasing the role of LGT on the evolution of a eukaryote.

## Results

**Fig. 1:**
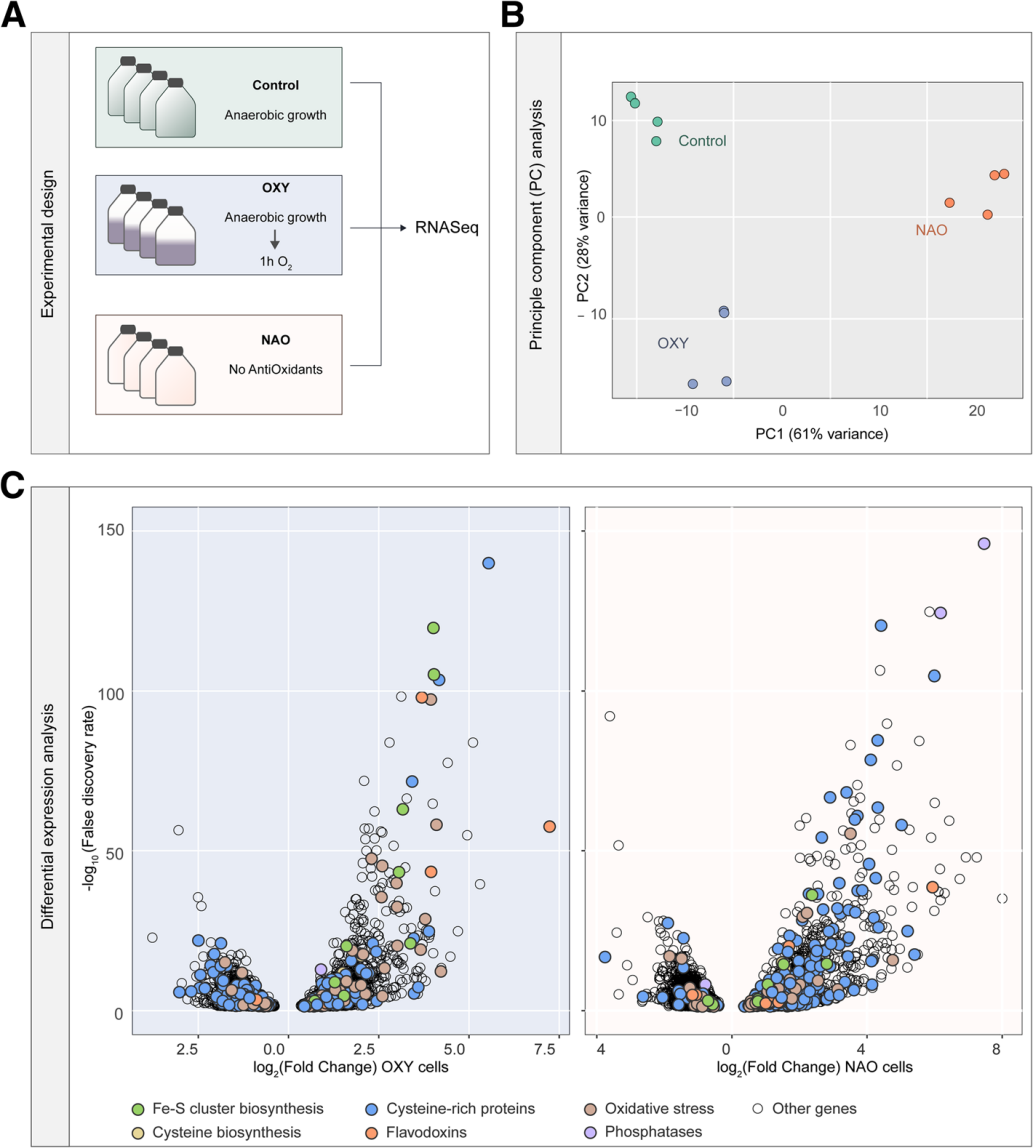
Experimental design and differential expression analysis of *S. salmonicida* exposed to oxygen and anti-oxidant deprivation. **a** RNA was isolated from cells grown to confluence anaerobically (green) and then exposed to oxygen for 1 h (OXY, purple) or grown to confluences without antioxidants (NAO, orange). **b** Four biological replicates for each condition separated into three defined clusters across 28% and 61% of variance of two principle components (PC). **c** Significantly differentially expressed genes from the OXY (left) and NAO (right) treatment conditions compared to the control anaerobic cells are shown as a function of their statistical significance (logarithmic of the adjusted *p* value (i.e., false discovery rate or *q* value)) and the magnitude of expression (logarithmic of the fold-change)

To investigate how *S. salmonicida* responds to oxygen and oxidative stress, cells were exposed to oxygen (OXY) or maintained in medium lacking antioxidants (NAO) (Fig. 1a, b). A total of 1031 and 674 genes were found to be up- and downregulated, respectively, in OXY-cells compared to controls, while 1424 and 856 genes were found to be up- and downregulated, respectively, in NAO-cells compared to controls (Fig. 1c, Additional file 1 and Additional file 2: Figure S1A). Between these two conditions, 623 and 225 genes were similarly up- and downregulated, respectively, and 41 and 8 genes displayed reciprocal regulation profiles (Additional file 2: Figure S1A). In both conditions, we observed within the top 100 upregulated genes, 45 and 59 genes, in OXY and NAO cells respectively, encode proteins of unknown function (Additional file 1). Similar patterns were seen in the top downregulated genes where most of the genes are predicted to encode hypothetical proteins (Additional file 3). We validated the relative expression values using qPCR for six target genes related to oxygen stress response (Additional file 1, Additional file 4: Figure S2). Below, we examine key pathways that were affected by these oxidative stress conditions and compare with similar gene expression studies in *G. intestinalis*.

### Oxygen and reactive oxygen species detoxification

Reactive oxygen species (ROS) are harmful species that derive from reactions with molecular oxygen. Unregulated changes to the steady-state levels of ROS can cause oxidative damage to proteins, lipids, and DNA (reviewed in [18, 19]). Eukaryotes use molecules (e.g., glutathione, carotenoids) or employ enzymes (e.g., Catalase, Superoxide dismutase) to clear these damaging compounds. However, diplomonads like *G. intestinalis* and *S. salmonicida* differ from other eukaryotes in that they lack many of these canonical eukaryotic defense strategies [16, 17]. Additionally, *S. salmonicida* does not encode enzymes necessary for glutathione biosynthesis. Below, we explore the gene expression profiles and evolutionary histories of the oxidative stress pathway in *G. intestinalis* and *S. salmonicida*.

#### Oxygen detoxification

**Fig. 2:**
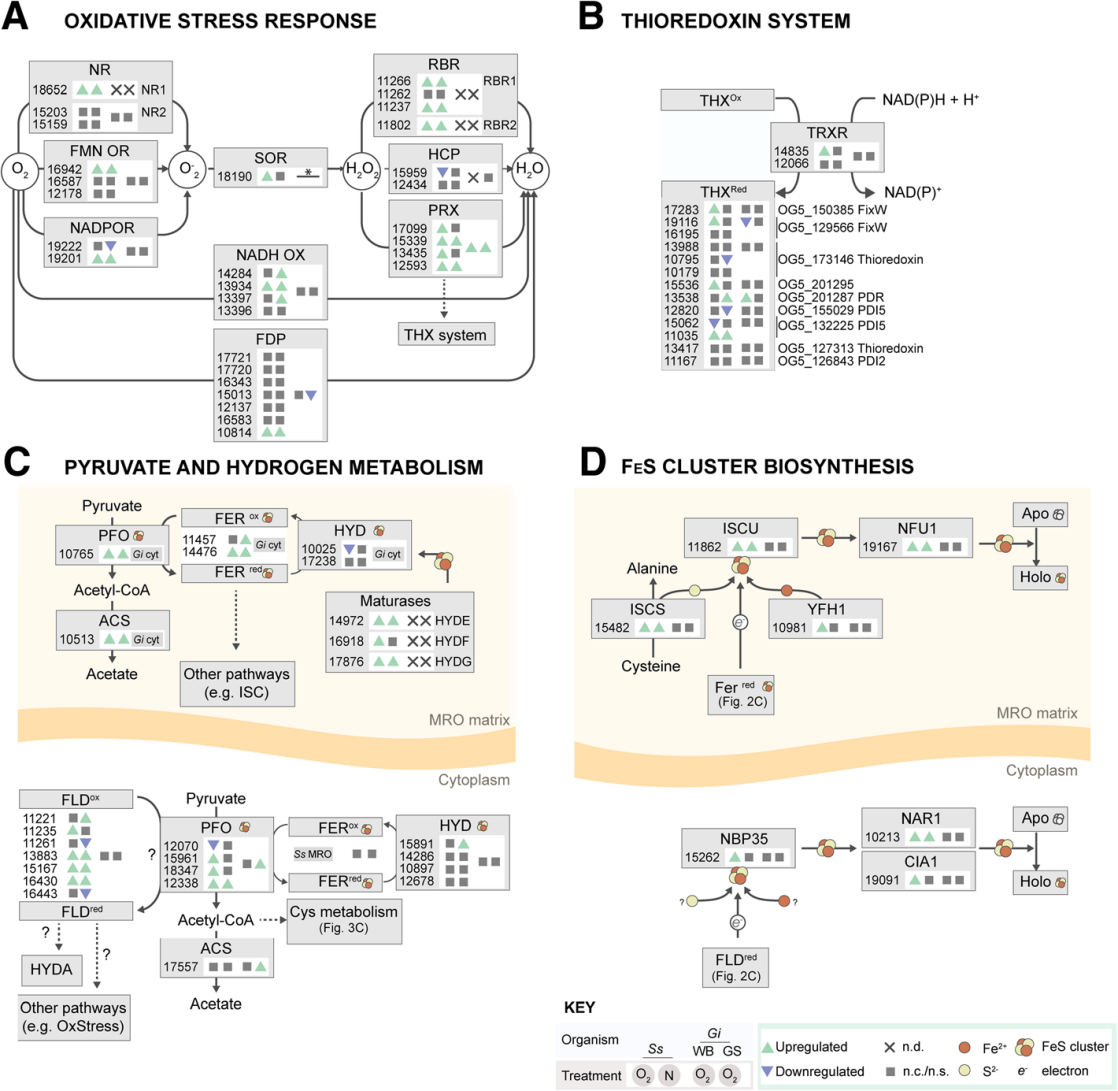
Genes encoding for proteins involved in **a** oxidative stress response, **b** thioredoxin recycling, **c** pyruvate metabolism, and **d** FeS cluster biosynthesis that are differentially expressed in OXY and/or NAO cells. See main text for detailed descriptions of each pathway and Tables 1 and 2 for abbreviations. For each *S. salmonicida* (*Ss*) gene, the *Giardia*DB gene accession number is indicated (SS50377_#####) and whether the gene is upregulated (up arrowhead), downregulated (down arrowhead), or unchanged (n.c.)/not significant (n.s.) (square) in OXY (first position) or NAO (second position) cells. When available, expression pattern for *G. intestinalis* (*Gi*) WB and GS assemblage in response to oxygen are shown in the third and fourth position respectively. “X” indicates that no homologue was detected (n.d.) in the *G. intestinalis* genome. Different sub-cellular localization patterns between *S. salmonicida* and *G. intestinalis* are shown in grey boxes. *G. instestinalis does encode an SOR homologue; however, the gene model was not predicted in previous projects, and therefore direct comparison of the gene expression was not possible

In *G. intestinalis*, oxygen can be directly converted to water by the redundant action of NADH oxidase (NADHox) [20] or Flavodiiron protein (FDP) [21, 22]. Three *S. salmonicida nadhox* genes are upregulated (Table 1, Fig. 2a) and only one of the seven FDP genes is upregulated in OXY and NAO cells. In *G. intestinalis*, oxygen can also be converted to a superoxide anion by a Ferredoxin-nitroreductase (FdNR1) [23], by a NADPH oxidoreductase (NADPH OR [17]), and potentially via an FMN-oxidoreductase (FMN-OR; InterPro id: IPR013785 [24]). In RNAseq expression studies of *G. intestinalis*, only the *fdnr1* gene is significantly upregulated in response to oxidative stress [25]; however, a microarray study also observed an increase in expression of *nadhox*, *nadphor*, and *fdnr1* genes in *G. intestinalis* cells exposed to one or more oxidative stressors [26]. *S. salmonicida* does not encode a homologue of the *G. intestinalis* FdNR1 protein, but instead two genes that share low sequence similarity (*nr1* and *nr2*; Fig. 2a). Collectively, genes encoding these oxygen-metabolizing proteins are upregulated in both OXY- and NAO-treated cells (*nr1*, SS50377_18652; *fmnor*, SS50377_16942; *NADPHOR*, SS50377_19201; Fig. 2a). The resulting superoxide from these reactions is then reduced to H_2_O_2_ by the action of a Superoxide reductase (SOR) [27]. The *S. salmonicida sor* gene (SS50377_18190) is upregulated in OXY- but not NAO-treated cells.

**Table 1:** Genes encoding proteins involved in the oxidative stress response that are differentially regulated in OXY and NAO *S. salmonicida* cells

Protein name	Abbr. In Fig. 2	OrthoMCL group	*Spironucleus salmonicida*				*Giardia intestinalis WB*		*Giardia intestinalis GS*	
			Gene ID (SS50377_)	NCBI accession	OXY LFC^a^	NAO LFC^a^	Gene ID (GL50803_)	LFC^b^	Gene ID (GL50581_)	LFC^b^
Flavodiiron protein	FDP	OG5_131036	10814	EST48966.1	2.0	1.1	10358	-	1626	- 0.9
FAD/FMN dependent oxidoreductase	FMN OR	OG5_127243	16942	EST43278.1	3.0	3.5	9719	-	2696	-
Hybrid cluster protein	HCP	OG5_133753	15959	EST44235.1	- 1.6	-	3042	-	-	-
NADH oxidase	NADHOX	OG5_126784	14284	EST45712.1	-	1.6	33769	-	2357	-
NADH oxidase	NADHOX	OG5_126784	13397	EST46593.1	-	1.5	33769	-	2357	-
NADH oxidase	NADHOX	OG5_126784	13934	EST45955.1	2.6	1.1	33769	-	2357	-
NADPH oxidoreductase	NADPHOR	OG5_130380	19222	EST41494.1	-	- 1.8	15004	-	2479	-
NADPH oxidoreductase	NADPHOR	OG5_130380	19201	EST41475.1	3.9	2.2	15004	-	2479	-
Nitroreductase	NR1	OG5_137896	18652	EST41818.1	3.7	2.5	c	c	c	c
Peroxiredoxin	PRX	OG5_126593	13435	EST46632.1	1.1	-	14521	1.0	2907	1.4
Peroxiredoxin	PRX	OG5_126593	17099	EST43234.1	1.8	-	16076	2.0	2907	1.4
Peroxiredoxin	PRX	OG5_126593	12593	EST47325.1	2.6	2.1	n/a	n/a	n/a	n/a
Peroxiredoxin	PRX	OG5_126593	15339	EST44769.1	3.8	2.0	n/a	n/a	n/a	n/a
Rubrerythrin 1	RBR1	OG5_132921	11266	EST48653.1	2.6	3.1	n/a	n/a	n/a	n/a
Rubrerythrin 1	RBR1	OG5_132921	11237	EST48625.1	4.2	4.8	n/a	n/a	n/a	n/a
Rubrerythrin 2	RBR2	OG5_132921	11802	EST48036.1	3.0	1.4	n/a	n/a	n/a	n/a
Superoxide reductase	SOR	OG5_144464	18190	EST42319.1	1.5	-	d	d	d	d
Thioredoxin	THX	OG5_132225	15062	EST45043.1	- 1.2	-	8064	-	3370	-
Thioredoxin	THX	OG5_132225	11035	EST48719.1	2.1	1.8	8064		3370	-
Thioredoxin	THX	OG5_129566	19116	EST41399.1	1.8	-	23888	- 0.8	3056	-
Thioredoxin	THX	OG5_150385	17283	EST43124.1	2.1	-	6289	-	4194	-
Thioredoxin	THX	OG5_155029	12820	EST47112.1	-	- 1.0	14670	-	3386	-
Thioredoxin	THX	OG5_173146	10795	EST48951.1	-	- 1.2	104250	-	661	-
Thioredoxin	THX	OG5_201295	15536	EST44537.1	1.3	-	9045	-	1623	-
Thioredoxin	THX	OG5_201287	13538	EST46454.1	-	1.1	9355	1.1	1609	-
Thioredoxin reductase	THXR	OG5_127532	14835	EST45259.1	2.3	-	9827	-	832	-
Carotenoid isomerase	-	OG5_131173	12033	EST47882.1	1.2	2.2	-	-	-	-
Carotenoid isomerase	-	OG5_131173	15222	EST44881.1	1.6	2.2	-	-	-	-

^a^|Log2 (fold change)| > 1.0 with q-values < 0.05 from this study; '-' indicates not significant changes

^b^|Log2 (fold change)| > 0.7 with p-values < 0.05 from Ma'ayeh et al. [25]; '-' indicates not significant changes

^c^*Giardia intestinalis* encodes a different orthologue of nitroreductase

^d^The *superoxide reductase* gene is not predicted in the most recent version of the Giardia instestinalis genome however has been identified in [27]

n/a indicates that the gene was not found in *Giardia intestinalis*

Since NADHox, FDP, and SOR are not part of the typical eukaryotic oxygen defense strategies, we explored the distribution and evolutionary history of these proteins in eukaryotes (Additional file 5: Figure S3 and Additional file 6). In general, genes encoding these proteins are rare in eukaryotes and were only found in transcriptome and genome sequencing projects of a handful of unicellular eukaryotes known to inhabit oxygen-poor environments (e.g., *Trichomonas vaginalis* [28], *Pygsuia biforma* [29], *Trepomonas* sp. [30], *Stygiella incarcerata* [31]). Phylogenetic analysis of NADHox demonstrates that diplomonads (*G. intestinalis*, *S. salmonicida*, and *Trepomonas* sp*.*) are monophyletic (Additional file 5: Figure S3A; Additional file 6 bootstrap value [BV] = 98) and emerge as a sister clade to a collection of, mostly Firmicute, bacteria with maximal support (Additional file 5: Figure S3A). This suggests that these genes were likely acquired by diplomonads from a prokaryotic donor by lateral gene transfer (LGT). Similarly, in the FDP phylogeny, the *G. intestinalis* and *S. salmonicida* sequences branch together with a collection with eukaryotic anaerobes (e.g., the breviate *Pygsuia biforma*, Trichomonads, and jakobid *Stygiella incarcerata*) and three bacteria sequences with maximal support sister to a clade of prokaryotes of mixed taxonomic classifications (Additional file 5: Figure S3B; Additional file 6). Finally, in the SOR phylogeny, *G. intestinalis*, *S. salmonicida*, and *Trepomonas* sp*.* sequences form a maximally supported clade adjacent to prokaryotic sequences (Additional file 5: Figure S3C). In all three cases, genes encoding these proteins were not detected in the vast majority of eukaryotic genomes and transcriptomes suggesting that these genes were likely acquired by LGT into the ancestor of diplomonads (e.g., *nadhox* and *sor*) or potentially metamonads (e.g., *fdp*).

#### Peroxide detoxification

In *G. intestinalis*, the resulting H_2_O_2_ is detoxified to water by the Peroxiredoxin (PRX) [32] with the help of an unknown redox partner. In other organisms, PRX interacts with the Thioredoxin (THX) system (Fig. 2b); however, attempts to measure *G. intestinalis* PRX activity coupled to *G. intestinalis* THX in vitro have been unsuccessful [32]. Nevertheless, we have depicted the PRX-THX system as one possible orientation for hydrogen peroxide detoxification (Fig. 2b). In microarray studies of *G. intestinalis*, genes encoding PRX and THX are both upregulated in response to one or more oxygen stressor [26]. In *S. salmonicida*, two *prx* genes are upregulated in OXY and NAO cells (SS50377_12593, SS50377_15339) or only OXY cells (SS50377_13435, SS50377_17099). We identified 13 genes encoding proteins containing a predicted thioredoxin domain, of which eight were differentially regulated in OXY and NAO cells (Fig. 2b, Table 1). We also identified a *thxr* gene (SS50377_14835) that is upregulated only in OXY cells. From these data alone, it is difficult to deduce which THX-containing proteins work together with PRX; however, we suspect that some of the genes encoding THX proteins (e.g., SS50377 15062, SS50377_12820, SS50377_10795) do not function in oxygen and oxidative stress given their downregulation in OXY or NAO cells.

In *S. salmonicida*, and not *G. intestinalis*, H_2_O_2_ can also be metabolized by Rubrerythrin (RBR). This Fe-containing protein has been implicated in the oxidative stress response of *Trichomonas vaginalis* [33], *Entamoeba histolytica* [34], and many bacterial pathogens [35, 36]. The redox partner of RBR has not been determined in *S. salmonicida* or other eukaryotes, though, in prokaryotes, some studies have speculated NADH could act as an electron donor [37, 38]. Two *rbr1* (SS50377_11266, SS50377_11237) and one *rbr2* (SS50377_11802) genes are among the highest differentially expressed transcripts in both OXY and NAO transcriptomes with LFC values ranging from 1.4 to 4.8 (Fig. 2a, Table 1).

Given that RBR are not found in most eukaryotes, we examined the evolutionary history of this protein*.* We identified genes encoding RBR in anaerobic amoebozoa (e.g., *Entamoeba histolytic* and *Mastigamoeba balamuthi*), Trichomonads, and *Trepomonas* sp. *S. salmonicida* and *Trepomonas* sp. sequences emerge as a monophyletic clade with maximal support (Additional file 5: Figure S3D, Additional file 6; grey circle) that is nested within a well-supported clade composed of bacteria with mixed taxonomic affinities (BV = 96; square label, Additional file 5: Figure S3D). This clade is separated from the Trichomonad clade, but this separation received only moderate support. The lack of representation of this gene across eukaryotic diversity, combined with the close relationship to bacteria, strongly suggests this gene was acquired by the ancestor of diplomonads, or potentially metamonads, from a bacterial donor.

#### Non-enzymatic ROS quenching

Carotenoids are small molecules responsible for protection against ROS [39] and are abundant in salmon [40]. Previous reports have demonstrated that *S. salmonicida* encodes a carotenoid isomerase protein (but no other proteins related to carotenoid biosynthesis), that was likely acquired from bacteria by LGT [41]. *S. salmonicida* encodes three non-identical copies of this gene, two of which (SS50377_12033 and SS50377_15222) were upregulated in OXY and NAO cells (Table 1).

We reconstructed the evolutionary history of carotenoid isomerase across the tree of life (Additional file 7: Figure S4A). We find that the *S. salmonicida* homologues emerge from a clade composed of animal pathogens, such as Spirochaetales (*Brachyspira* species) and Campylobacterales (*Helicobacter* and *Campylobacter* species), with maximal support (Additional file 7: Figure S4A). In these bacterial genomes, we manually examined the genetic neighborhood of the carotenoid isomerase gene to other genes encoding proteins related to carotenoid metabolism. While there was no consistent operon structure in the closest bacterial homologues that would allow inference of cartenoid metabolism, we observed that there were genes encoding for oxidative stress response proteins (Additional file 7: Figure S4A). For example, in some species of *Brachyspira*, carotenoid isomerase is adjacent to genes encoding Flavodoxin, NADHox, and/or Ferritin (Additional file 7: Figure S4A). Unfortunately, little is known about the function of this type of carotenoid isomerase in prokaryotes so we are unable to predict its precise role in *S. salmonicida*.

To determine the subcellular localization of this protein, we transfected *S. salmonicida* cells with the SS50377_15222 gene tagged with an OLLAS tag. Antibodies raised against the OLLAS tag show similar distribution patterns for endoplasmic reticulum (ER) localization (Additional file 7: Figure S4B) shown previously (Sec61 localization Figure 4a in [42] and ultrastructure studies Figure 9 in [9]). Other *S. salmonicida* proteins related to oxygen stress response such as, protein disulfide isomerase, have previously been shown to localize to the ER [42] which is congruent with reports emphasizing the role of the ER in the oxygen stress response in eukaryotes [43].

### Pyruvate and hydrogen metabolism

In general, genes encoding proteins that function in the hydrogenosome are upregulated in both OXY and NAO cells (Fig. 2c, Table 2). More specifically, genes encoding hydrogenosomally localized PFO (SS50377_10765), FER (SS50377_14476 and SS50377_11457), and ACS (SS50377_10513) are upregulated in OXY and NAO cells. A similar pattern was seen for the genes encoding cytoplasmic PFO in OXY and NAO cells (Fig. 2c, Table 2). In the absence of a cytoplasm-localized FER, we speculate that one of the seven short-chain flavodoxin-related proteins (FLD) encoded by *S. salmonicida* [17] could serve as the electron carrier for PFO. This is in agreement with studies of bacteria that have demonstrated PFO can reduce FLDs [44] as well as FER. Like PFO, five of these FLD genes are upregulated in both OXY and NAO cells with log_2_fold changes as high as 7.2 and 5.9 respectively (Fig. 2c, Table 2).

**Table 2:** Genes encoding proteins involved in hydrogen and pyruvate metabolism that are differentially regulated in OXY and NAO *S. salmonicida* cells

Protein name	Abbr. In Fig. 2	OrthoMCL group	*Spironucleus salmonicida*				*Giardia intestinalis WB*		*Giardia intestinalis GS*	
			Gene ID (SS50377_)	NCBI accession	OXY LFC^a^	NAO LFC^a^	Gene ID (GL50803_)	LFC^b^	Gene ID (GL50581_)	LFC^b^
[2Fe-2S] Ferredoxin 1	FER	OG5_126994	14776	EST45204.1	3.0	1.8	27266	-	3971	-
[2Fe-2S] Ferredoxin 2	FER	OG5_126994	11457	EST48363.1	-	1.1	27266	-	3971	-
Pyruvate:ferredoxin oxidoreductase 1	PFO	OG5_129721	12338	EST47643.1	4.1	1.7	114609	0.8	1047	-
Pyruvate:ferredoxin oxidoreductase 2	PFO	OG5_129721	18347	EST42040.1	1.6	-	114609	0.8	1047	-
Pyruvate:ferredoxin oxidoreductase 3	PFO	OG5_129721	15961	EST44237.1	1.4	-	114609	0.8	1047	-
Pyruvate:ferredoxin oxidoreductase 4	PFO	OG5_129721	12070	EST47847.1	- 1.8	-	114609	0.8	1047	-
Pyruvate:ferredoxin oxidoreductase 5	PFO	OG5_129721	10765	EST48995.1	2.0	1.2	114609	0.8	1047	-
[FeFe]-hydrogenase 4	HYDA	OG5_127303	15891	EST44288.1	-	1.9	6304	-	4021	-
[FeFe]-hydrogenase 6	HYDA	OG5_127303	10025	EST49607.1	- 3.0	-	6304	-	4021	-
HYDA assembly protein HydE	HYDE	OG5_155498	14972	EST44954.1	1.5	1.5	n/a	n/a	n/a	n/a
HYDA assembly protein HydF	HYDF	OG5_168779	16918	EST43253.1	2.8	-	n/a	n/a	n/a	n/a
HYDA assembly protein HydG	HYDG	OG5_139637	17876	EST42561.1	3.3	1.4	n/a	n/a	n/a	n/a
Acetyl-CoA synthetase	ACS	OG5_131604	10513	EST49290.1	1.5	1.4	13608	1.2	1568	-
Flavodoxin	FLD	OG5_127162	11261	EST48648.1	-	- 1.1	15897	-	735	-
Flavodoxin	FLD	OG5_127162	16443	EST43822.1	-	- 1.2	15897	-	735	-
Flavodoxin	FLD	OG5_127162	11221	EST48609.1	-	1.4	15897	-	735	-
Flavodoxin	FLD	OG5_127162	11235	EST48623.1	1.0	-	15897	-	735	-
Flavodoxin	FLD	OG5_127162	16430	EST43810.1	3.7	1.7	15897	-	735	-
Flavodoxin	FLD	OG5_127162	15167	EST45144.1	3.9	1.3	15897	-	735	-
Flavodoxin	FLD	OG5_127162	13883	EST45907.1	7.2	5.9	15897	-	735	-

^a^|Log2 (fold change)| > 1.0 with q-values < 0.05 from this study; '-' indicates not significant changes

^b^|Log2 (fold change)| > 0.7 with p-values < 0.05 from [25]; '-' indicates not significant changes

n/a indicates that the gene was not found in *Giardia intestinalis*

Unlike pyruvate metabolism genes, the expression of genes encoding hydrogenosomally localized HYD (SS50377_17238, SS50377_10025) are not upregulated in OXY and NAO cells despite significant upregulation of genes encoding the HYD maturases (Table 2). Similarly, most of the genes encoding cytoplasmic HYD protein (Fig. 2c) had expression values below our significance thresholds (Additional file 1) except the *hyd4* (SS50377_15891) gene which was significantly upregulated in NAO cells.

### Fe-S cluster biosynthesis and Fe-S cluster proteins

Iron-sulfur clusters are essential biological cofactors that participate in a variety of electron transfer reactions (for review, see [45]). Many Fe-S cluster proteins and sensitive to oxygen [46, 47] and require repair or proteosomal clearance once damaged (discussed below). Fe-S cluster proteins also play a critical role in the repair of DNA damage [48], a cellular consequence of oxidative stress [49]. In most mitochondria and related organelles, the iron-sulfur cluster (ISC) system is responsible for the maturation of mitochondrial Fe-S clusters [50]. Genes encoding the core components of ISC machinery that function in the hydrogenosome are upregulated in OXY and NAO cells (Fig. 2d, Table 3). In the cytoplasm, Fe-S clusters are assembled via the cytoplasmic iron-sulfur cluster assembly (CIA) pathway. Previous studies identified genes encoding three CIA components in the genome of *S. salmonicida* [51]: the scaffolding protein (*nbp35*) and chaperones (*nar1* and *cia1*). Genes encoding NBP35 and CIA1 are upregulated in OXY cells but not NAO cells (Fig. 2d, Table 3). We next surveyed the genome for genes encoding Fe-S cluster or Fe-binding proteins using MetalPredator [52]. We identified 60 genes encoding putative Fe-S cluster proteins, 41 of which were significantly differentially expressed in OXY and/or NAO cells (Fig. 2d, Table 3). Most of these genes were upregulated including a gene encoding DNA damage repair helicase (SS50377_10417; Table 3).

**Table 3:** Genes encoding FeS cluster proteins and FeS cluster biosynthesis proteins that are differentially regulated in OXY and NAO Spironucleus cells

Protein name	Abbr. In Fig. 2, 3	FeS cluster type	OrthoMCL group	*Spironucleus salmonicida*				*Giardia intestinalis WB*		*Giardia intestinalis GS*	
				Gene ID (SS50377_)	NCBI accession	OXY LFC^a^	NAO LFC^a^	Gene ID (GL50803_)	LFC^b^	Gene ID (GL50581_)	LFC^b^
*Fe-S cluster biosynthesis*											
Cysteine desulfurase	ISCS	c	OG5_126959	15482	EST44485.1	4.0	2.4	14519	-	3589	-
Frataxin	YFH1	c	OG5_128372	10981	EST48875.1	1.5	-	n/a	n/a	n/a	n/a
FeS cluster scaffold protein	ISCU	c	OG5_127020	11862	EST48017.1	3.2	1.5	15196	-	3674	-
NifU-like protein	NFU1	c	OG5_127523	19167	EST41448.1	4.0	1.1	32838	-	1173	-
FeS cluster assembly factor	NAR1	c	OG5_196623	10213	EST49464.1	-	-	33030	-	2775	-
Cytosolic FeSprotein assembly	CIA1	c	OG5_127906	19091	EST41375.1	1.6	-	17550	-	1833	-
Nucleotide-binding protein	NBP35	c	OG5_126620	15262	EST44817.1	3.1	-	10969	-	2003	-
*Fe-S cluster proteins*											
Diphthamide biosynthesis protein	-	4Fe-4S	OG5_127598	12464	EST47479.1	1.7	-	4248	-	3444	-
DNA polymerase	-	4Fe-4S	OG5_128651	13679	EST46293.1	-	1.4	n/a	n/a	n/a	n/a
DNA repair helicase	-	4Fe-4S	OG5_127294	13430	EST46627.1	1.3	1.5	5631	-	3240	-
DNA repair helicase RAD3	-	4Fe-4S	OG5_127585	10417	EST49200.1	1.3	1.4	4328	-	2184	-
DNA polymerase alpha	-	4Fe-4S	OG5_127945	14337	EST45766.1	-	1.3	6980	-	4344	-
RNA polymerase RPB3	-	4Fe-4S	OG5_127622	14437	EST45591.1	-	- 1.1	7474	-	2249	-
RNA polymerases I and III	-	4Fe-4S	OG5_128425	19186	EST41464.1	-	- 1.9	10840	-	1230	- 0.8
Cysteine-rich protein	-	4Fe-4S	OG5_159029	16132	EST44066.1	-	- 1.5	10329	-	4058	-
Ferredoxin Fd3	-	4Fe-4S	OG5_159029	13915	EST45936.1	1.4	1.0	10329	-	4058	-
Ferredoxin	-	4Fe-4S	OG5_136509	17123	EST43182.1	- 2.2	- 1.3	n/a	n/a	n/a	n/a
Glutamate synthase	-	4Fe-4S	OG5_126892	19044	EST41331.1	-	1.5	7195	-	3819	-
Putative ferredoxin	-	4Fe-4S	OG5_126892	17234	EST43076.1	-	- 1.1	7195	-	3819	-
Putative ferrodoxin	-	4Fe-4S	OG5_187401	10151	EST49544.1	- 2.1	- 2.0	n/a	n/a	n/a	n/a
Radical SAM superfamily protein	-	4Fe-4S	OG5_138560	12417	EST47432.1	2.3	-	16519	-	358	0.7
Histone acetyltransferase Elp3	-	4Fe-4S	OG5_127324	10392	EST49177.1	2.9	2.5	16639	-	2520	-
Glycerol-3-phosphate dehydrogenase	-	2Fe-2S	OG5_127956	15333	EST44764.1	-	1.1	16125	-	2252	-
Molybdenum cofactor sulfurase	-	2Fe-2S	OG5_129340	18322	EST42015.1	1.7	1.6	14200	-	2926	-
Selenophosphate synthase	SPS-NifS	?	OG5_131755	16780	EST43726.1	1.5	1.6	n/a	n/a	n/a	n/a
hypothetical protein	-	?	EIN_200440	18400	EST42092.1	-	1.9	n/a	n/a	n/a	n/a

^a^|Log2 (fold change)| > 1.0 with q-values < 0.05 from this study; '-' indicates not significant changes

^b^|Log2 (fold change)| > 0.7 with p-values < 0.05 from Ma'ayeh et al. [25]; '-' indicates not significant changes

^c^transiently binds Fe-S clusters

n/a indicates that the gene was not found in *Giardia intestinalis*

### Cysteine-rich proteins, cysteine metabolism, and methionine repair

**Fig. 3:**
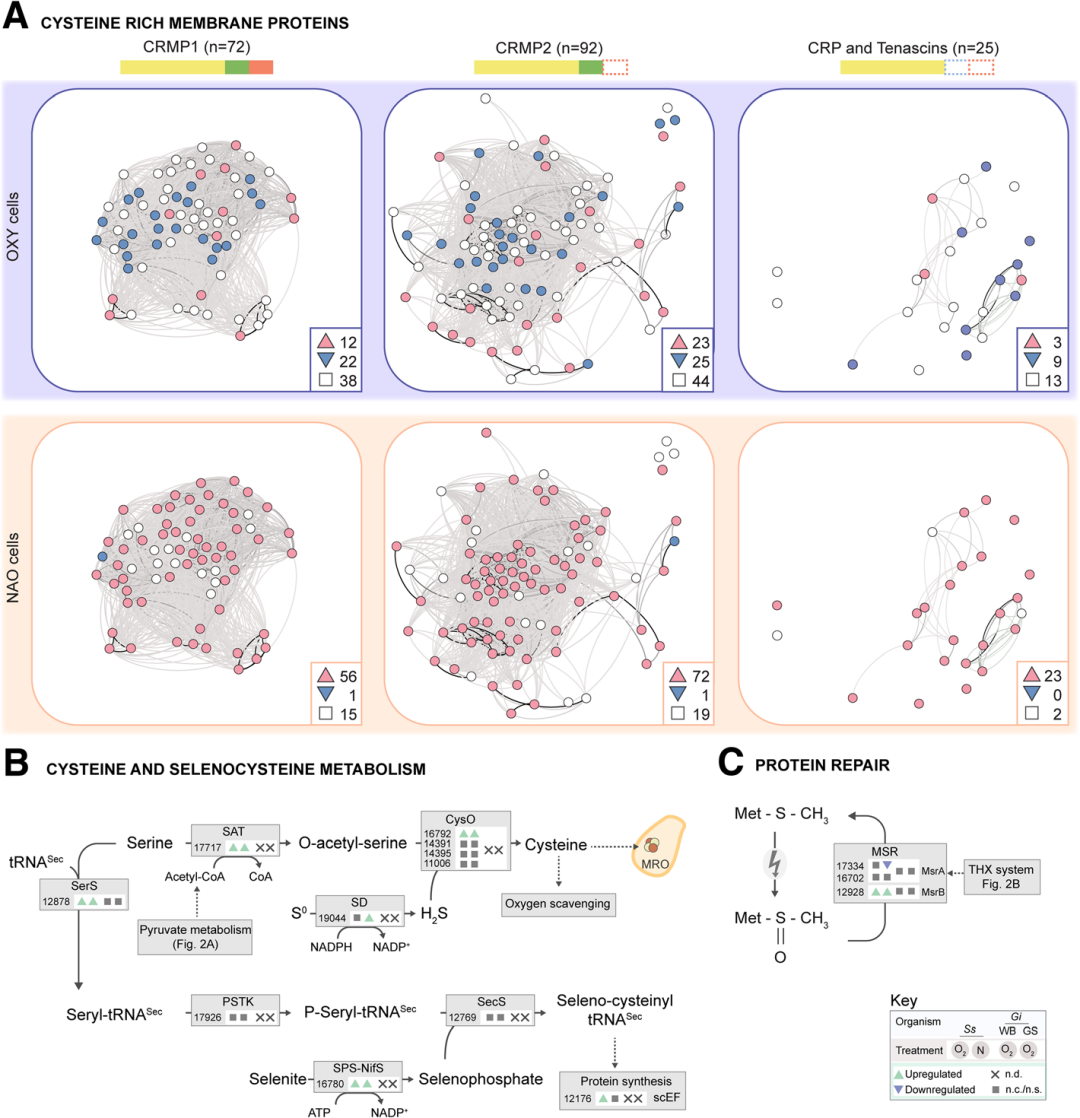
Cysteine-associated processes are differentially expressed in response in OXY and NAO *S. salmonicida* cells. **a** Evolutionary gene network of all differentially expressed genes encoding cysteine-rich proteins (CRPs and Tenascins) and membrane proteins (CRMP1 and CRMP1) in OXY (purple) and/or NAO (orange) cells. Each node represents one cysteine-rich protein where edges were weighted by pair-wise sequence identity. Proteins are subclassified is based on their domain composition into region rich in CXC and CXXC domains (yellow), transmembrane domain (green) and conserved [KR][KR]X[KR][KR] motif (red) as indicated. Gene nodes with log_2_fold changes greater than or less than 1.0 are shown in red and blue respectively; genes with non-significant expression changes are shown in white; numerical summary for upregulated (up arrowhead), downregulated (down arrowhead) and non-significant (square) genes are shown. **b** Cysteine is synthesized from serine and acetyl-CoA via serine acetyl transferase (SAT) to O-acetyl-serine which is thiolated by cysteine synthase (CysO) using sulfide derived from elemental sulfur via sulfide dehydrogenase (SD). Selenocysteine is synthesized directly on a serine-charged selenocysteine tRNA. Serine is added to the tRNA by Seryl-tRNA synthetase (SerS) and phosphorylated by L-Seryl-tRNA(Sec) kinase (PSTK). Selenophosphate, derived from selenite (or selenocysteine, not shown), is generated via Phosphoseryl-tRNA(Sec) selenium transferase (SPS), is added via Selenophosphate synthetase, cysteine desulfurase fusion protein (SecS-NifS). Pathway labeling is as described in Fig. 1 and summarized in key. **c** Repair of oxygen-damaged methionine residues in proteins. MSR peptide methionine sulfoxide reductase, THX thioredoxin

*G. intestinalis* possesses hundreds of genes encoding variant surface proteins (VSPs) that provide antigenic variation to avoid the host immune system [53]. These proteins have an average cysteine content of 12% and often contain a conserved C-terminal sequence motif (CRGKA). *S. salmonicida* encodes a similar set of cysteine-rich proteins that were further sub-classified into three categories (detailed in Fig. 3a) [17]. Of these 385 genes, 189 were differentially expressed in one or both conditions (Fig. 3a). In fact, these genes were among the most upregulated genes in the transcriptome (Additional file 1). We observed that in OXY cells, the gene expression of most cysteine-rich proteins was unchanged or downregulated, while in the NAO cells, most cysteine-rich proteins were upregulated (Fig. 3a). Given the known role of cysteine in protecting anaerobes, such as *G. intestinalis*, from oxidative stress in vitro [54–56] and the number of cysteine-rich proteins expressed in *S. salmonicida*, we investigated the gene expression profiles of proteins responsible for the biosynthesis of cysteine in OXY and NAO cells. *S. salmonicida*, but not *G. intestinalis*, encodes genes for *de novo* biosynthesis of cysteine [17], and these genes are upregulated in both OXY and NAO cells (Fig. 3b, Table 4).

**Table 4:** Genes encoding cysteine and selenocysteine biosynthesis proteins that are differentially expressed in OXY or NAO *S. salmonicida* cells

Protein name	Abbr. In Fig. 3	OrthoMCL group	*Spironucleus salmonicida*				*Giardia intestinalis WB*		*Giardia intestinalis GS*	
			Gene ID (SS50377_)	NCBI accession	OXY LFC^a^	NAO LFC^a^	Gene ID (GL50803_)	LFC^b^	Gene ID (GL50581_)	LFC^b^
Cystathionine β-lyase	-	OG5_135774	11958	EST47938.1	1.6	1.0	n/a	n/a	n/a	n/a
Cysteine synthase	CS	OG5_126662	16792	EST43431.1	2.0	1.3	n/a	n/a	n/a	n/a
Peptide methionine sulfoxide reductase A	MSRA	OG5_126847	17334	EST43031.1	-	- 1.0	n/a	n/a	n/a	n/a
Peptide methionine sulfoxide reductase B	MSRB	OG5_127019	12928	EST46975.1	2.7	1.9	n/a	n/a	n/a	n/a
Selenocysteine-specific elongation factor	scEF	OG5_130322	12176	EST47777.1	1.1	-	n/a	n/a	n/a	n/a
Selenophosphate synthase, NifS fusion	SPS	OG5_131755	16780	EST43726.1	1.5	1.6	n/a	n/a	n/a	n/a
Selenoprotein W	-	OG5_134745	fx081	EST46492.1	-	- 1.0	8394	-	4139	-
Selenoprotein W	-	SS50377_fx080	fx080	EST46486.1	- 1.4	-	n/a	n/a	n/a	n/a
Serine acetyltransferase	SAT	OG5_128713	17717	EST42696.1	1.4	1.4	n/a	n/a	n/a	n/a
Seryl-tRNA synthetase	SerS	OG5_126677	12878	EST47068.1	1.2	2.0	101501	-	4129	-
Sulfide dehydrogenase	SD	OG5_126892	19044	EST41331.1	-	1.5	7195	-	3819	-

^a^|Log2 (fold change)| > 1.0 with q-values < 0.05 from this study; '-' indicates not significant changes

^b^|Log2 (fold change)| > 0.7 with p-values < 0.05 from Ma'ayeh et al. [25]; '-' indicates not significant changes

n/a indicates that the gene was not found in *Giardia intestinalis*

In other organisms, some oxygen detoxification proteins (e.g., glutathione peroxidase [57] or thioredoxin reductase [58]) contain the non-conventional amino acid selenocysteine which is an effective reducing agent under oxidative stress [59]. This amino acid is incorporated into proteins co-translationally using a selenocysteine-charged tRNA (tRNASec) and dedicated selenocysteine elongation factor (scEF) that together recognize UGA. The *S. salmonicida* genome encodes the necessary machinery for the synthesis of selenocysteine-charged tRNA and protein elongation [17] (Fig. 3b) and at least three selenoproteins (Table 4). Despite selenocysteine’s predicted role in oxygen defense, most of the genes encoding selenocysteine biosynthesis proteins and selenoproteins were not upregulated (Fig. 3b, Table 4); however, we did see upregulation of genes involved in selenophosphate generation in OXY and NAO cells and protein elongation in NAO cells.

ROS can also damage methionine residues in proteins by forming a methionine sulfoxide [60]. To repair this damage, many organisms use peptide methionine sulfoxide reductase (MSR) A and B depending on the stereochemistry of the oxidation event using electrons derived from the thioredoxin system [61] (Fig. 3c, Table 4). MSR proteins are also found in many bacteria where they play an important role in the defense against oxidative stress [62–65]. We observed that the *msrB* gene is upregulated in both OXY and NAO cells. However, *msrA* gene expression appears to be unaffected in OXY cells or in fact downregulated in NAO cells (Additional file 1 and Additional file 4: Figure S2).

### Hypothetical proteins

Perhaps, one of the most unexpected findings from the *S. salmonicida* genome was the detection of 4903 genes encoding for hypothetical proteins [17]. These hypothetical proteins are classified as “conserved” (847 genes; 812 non-identical gene copies) or “*S. salmonicida*-specific” (4057 genes; 3729 non-identical gene copies) based on whether they have detectable homologues in other organisms. In both the conserved and *S. salmonicida*-specific hypothetical protein sets, only a fraction of proteins contained detectable interpro domains (48% and 7% respectively; Additional file 3). However, a larger fraction of proteins from the *S. salmonicida*-specific hypothetical protein set have predicted transmembrane domains compared to the conserved hypothetical protein set (9% and 0.4%, respectively; Additional file 3). Bioinformatic prediction of the subcellular localization of non-model organisms such as *S. salmonicida* is hindered by the lack of reliable tools. Nevertheless, we predicted the localization of all *S. salmonicida* proteins using two prediction tools (BUSCA and DeepLoc; Additional file 3), although these predictions should be interpreted with caution. Of the conserved hypothetical proteins, 184 and 243 genes were differentially expressed in OXY and NAO cells, respectively (Additional file 2: Figure S1B; 61 and 36 of which were similarly up- or downregulated, respectively). We further compared a 360-gene subset of these hypothetical proteins that are only found in *S. salmonicida* and *G. intestinalis* where 82 and 99 genes were differentially expressed in OXY and NAO cells respectively (Additional file 2: Figure S1C). While we cannot conclusively determine the function of these proteins, at least one of the upregulated genes encodes for a thioredoxin-domain containing protein (Table 3; SS50377_13538). In terms of the *S. salmonicida*-specific genes, 896 and 1221 genes were differentially expressed in OXY and NAO cells respectively (Additional file 2: Figure S1D).

### Host-microbe interaction

As *S. salmonicida* approaches the epithelium of the host, the relative concentration of oxygen increases [66], and therefore, oxygen might act as an aerotactic signal for pathogenicity [67]. To gain access of the circulatory system of salmon, *S. salmonicida* likely relies on proteases to degrade the host epithelium like other pathogens [68]. *S. salmonicida* encodes at least 111 proteases most of which belong to four main classes: threonine, serine, metallo, and cysteine proteases [17]. In OXY cells, more than half of the threonine (12/14) and metalloproteases (17/33) encoded in the genome were upregulated (Additional file 8: Figure S5A). Many of these OXY-upregulated proteases are components of the proteasome (Additional file 8: Figure S5A). In some eukaryotes, including parasites, the proteasome is upregulated in response to oxygen stress to allow for rapid turnover of oxygen-damaged proteins [69, 70] and has even been found to be essential for development during oxygen stress in some parasites [71]. How these genes are regulated in response to oxygen is well documented in model organisms [69] and involves the concerted effort of multiple transcription factors in yeast (Yap1p, Rpn4p) and mammals (nuclear factor E2-related factors; NRF1, NRF2); however, we failed to detect homologues of these proteins the *S. salmonicida* genome suggesting that there must be an alternative set of transcriptional regulators for proteasome maintenance. Most of the proteasome was not upregulated in NAO cells suggesting that thiol stress does not produce as many oxygen-damaged proteins as oxygen exposure. In both OXY and NAO cells, many of the cysteine proteases were upregulated, while serine protease expression remained largely unchanged.

We also identified a number of other effector proteins such as the toxin hemolysin, efflux pumps, and secreted proteins that might play a role in *Spironucleus*-host or *Spironucleus*-microbiome interactions (Additional files 6 and 9).

### Other pathways

We also examined the gene expression patterns of transcription factors (Additional file 10: Figure S6), encystation-related proteins (Additional file 9), and gene families that have been expanded in *S. salmonicida* compared to (Additional file 11: Figure S7). Detailed discussion can be found in Additional file 9.

## Discussion

### Pyruvate and hydrogen metabolism

The energy metabolism of *S. salmonicida* relies on oxygen-sensitive proteins such as PFO [72] and FER [73]. Genes encoding these enzymes were upregulated in *S. salmonicida* OXY and NAO cells (Fig. 2c), suggesting the *S. salmonicida* PFO proteins could be oxygen-insensitive. While oxygen-insensitivity conferred by a C-terminal extension on the PFO protein has been reported in some bacteria [74, 75], we were unable to detect similar extensions in the *S. salmonicida* proteins, suggesting that these proteins are protected from oxidative damage by another mechanism. Indeed, oxygen-mediated inhibition of *Entamoeba histolytica* PFO has previously been shown to be partially or fully prevented by high levels of CoA or acetyl-CoA [76]. Furthermore, in *Bacteroides thetaiotaomicron,* oxygen-induced inactivation of PFO can be partially restored after transition to anaerobiosis for 40 min [77] suggesting that oxygen damage might not be permanent. Others have also reported that PFO inactivation can be partially prevented in *Campylobacter jejuni* by the action of unknown protective proteins specific for adaptation to oxygen transition zones [78]. Therefore, it is possible that there are alternative strategies for the *S. salmonicida* proteins to maintain partial enzymatic activity in oxygen, and therefore, oxygen levels do not reduce gene expression. Furthermore, the robust oxygen defense system of *S. salmonicida* might be able to maintain low intracellular oxygen concentrations even under direct oxygen exposure allowing for unperturbed enzymatic activity.

Assuming that the PFO proteins are functional in these oxidative stress conditions, the resulting reduced electron carrier and acetyl-CoA are predicted to have different fates depending on the subcellular location. Under anaerobic conditions, the reduced FER generated by hydrogenosomal PFO is re-oxidized by HYD to generate hydrogen. However, in OXY and NAO-treated cells, hydrogenosomal *hyd* gene expression is not affected, and therefore, the PFO-derived reduced FER might be re-oxidized by another system, such as the Fe-S cluster biosynthesis machinery.

In bacteria, FLDs have numerous roles including interacting with a FLD(FER):NAD(P)+ reductase (reviewed in [79]). In *G. intestinalis*, PFO-FER system has been shown to function in oxygen detoxification together with a FER:NAD(P)+ oxidoreductase activity and oxygen consumption [80] potentially catalyzed by a FDP. Taken together, it is possible that the cytoplasmic PFO-FLD system of *S. salmonicida* could provide electrons for oxygen clearance by FDP in OXY cells. The resulting acetyl-CoA from this cytoplasmic reaction might not participate in energy generation since the gene encoding cytoplasmic ACS was not upregulated. Instead, we propose that acetyl-CoA is used for the biosynthesis of cysteine as another line of defense against oxygen (Fig. 3b).

### ROS clearance

In general, we observed the ROS detoxification systems in *S. salmonicida* are similar to those seen in *G. intestinalis*. These organisms have surprisingly redundant strategies for some reactions, such as the conversion of oxygen to oxyanion, having as many as four different enzymes catalyzing the same reaction in *S. salmonicida* (Fig. 2a). It is difficult to speculate why this is; it could be that the reduction potentials, substrate affinity, and redox partners of these enzymes are each optimized for certain oxidative stress conditions. In *S. salmonicida*, and not *G. intestinalis*, we observed that many genes in the ROS response system are encoded in multiple non-identical copies consistent with the observed *S. salmonicida* gene expansion compared to *G. intestinalis* (Additional file 11: Figure S7).

**Fig. 4:**
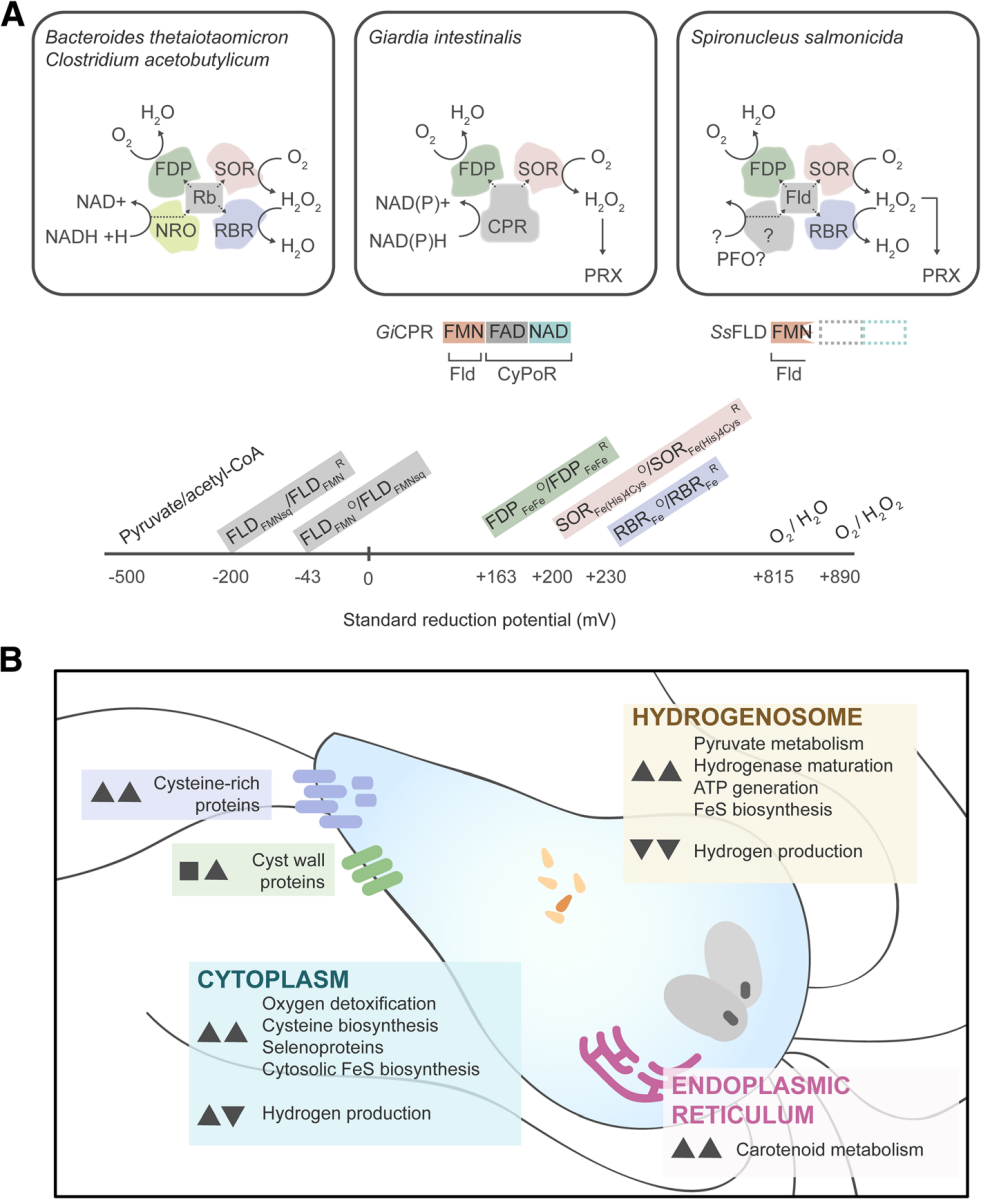
**a** Proposed multienzyme complex for ROS detoxification in bacteria (left), *G. intestinalis* (middle) and *S. salmonicida* (right). For *G. intestinalis and S. salmonicida,* Cytochrome p450 reductase (CPR) domain compositions are shown below the panel. In some strict anaerobic bacteria (left), electrons from NADH are transferred to the electron carrier rubredoxin (Rb) via NADH:Rb oxidoreductase (NRO) and ultimately to oxygen or hydrogen peroxide by the concerted action of flavodiiron protein (FDP), superoxide reductase (SOR), or rubrerythrin (RBR). In *G. intestinalis*, we propose that the CPR protein can funnel electrons from NAD(P)H to FDP or SOR ultimately reducing oxygen. In *S. salmonicida*, we propose a hybrid of the bacterial and *G. intestinalis* systems whereby a truncated CPR protein (i.e., the FLD domain; *Ss*FLD) functions as the electron carrier between FDP, SOR, and RBR. This hypothesis is supported by the observed reduction potentials of the proposed reactions shown in the reduction potential graph. **b** Summary of cellular functions affected by oxygen and antioxidant depletion. Next to each component, the observed gene regulation of OXY (left) and NAO (right) are shown as up arrowheads, down arrowheads, or squares to represent upregulated, downregulated, or unchanged genes expression respectively

*S. salmonicida*, and not *G. intestinalis*, encodes a gene for Rubrerythrin, an Fe-binding protein with peroxidase activity and is related to the Ferritin superfamily. *S. salmonicida* encodes multiple *rbr* genes which are upregulated in response to oxygen, although the electron donors for the RBR reaction are unknown. In some sulfate reducers and obligate anaerobes, RBR works together with SOR, FDP, rubredoxin (Rb), and an NADH:Rb oxidoreductase (NRO) in a five-member multi-enzyme complex [81] (Fig. 4a) with high affinities for oxygen (Km = 2.9 μM) and hydrogen peroxide (Km < 1 μM) [82]. This oxygen-inducible system helps maintain the anoxic environment for these obligate anaerobes. Similar co-operative systems between SOR and FDP and non-Rb electron carriers have also been observed [83]. We suspect that an analogous multi-enzyme complex could be at work in *G. intestinalis* and *S. salmonicida*. One such configuration in *G. intestinalis* could involve the SOR, FDP, and a pyridine dinucleotide oxidoreductase such as the soluble cytochrome P450 reductase (CPR) protein (Fig. 4a). CPR proteins have a CysJ domain that has been implicated in a wide range of electron transfer reactions whereby electrons derived from NADPH can donate to electron acceptors other than cytochrome P450 [84–86] which is especially evident in their identification in eukaryotes without cytochrome P450 such as *Blastocystis* sp. [87]. The FLD protein in *S. salmonicida* is in fact homologous to the N-terminal region of the *G. intestinalis* CPR protein (based on sequence similarity to those proteins reported [51]), but lacks the FAD and pyridine dinucleotide oxidoreductase (NAD) domain (Fig. 4a), suggesting that this protein alone cannot function in NAD(P)H oxidation. Instead, we propose that *S. salmonicida* uses a system that uses RBR, SOR, and FDP together with FLD and an FLD oxidoreductase such as PFO; similar systems have been proposed in bacteria [85]. Given the rather low reduction potential of pyruvate to acetyl-CoA (− 500 mV [88]), these reactions can readily be coupled to FLD reduction (− 280 mV [89]). These electrons can be ferried to the redox centers of one of three proteins present in *S. salmonicida* with favorable reduction potentials of + 163 mV, + 100–200 mV, and + 230 mV for FDP [22], SOR [90], and RBR [91] respectively (estimated from other organisms; Fig. 4b). Future experimental work with *S. salmonicida* will help us to confirm or refute this hypothesis.

Carotenoids are another powerful molecule for quenching ROS [39]. The presence of a gene encoding carotenoid isomerase-like protein suggests that *S. salmonicida* is able to metabolize carotenoids. However, the lack of genes encoding proteins involved in the *de novo* synthesis of carotenoids suggests that *S. salmonicida* might act on exogenously produced carotenoid precursors. Carotenoids are abundant in the diet of Atlantic salmon and are responsible for the characteristic pink flesh of salmonids [40] and therefore would be readily accessible for *S. salmonicida* during localized gut and systemic infections. Phylogenetic analysis suggests that this gene encoding carotenoid isomerase was laterally acquired by *S. salmonicida* from a Spirochaete-like donor (Additional file 7: Figure S4A). Most of the bacteria that branch closely to *S. salmonicida* in this phylogeny have been isolated from the gut or fecal material of animals as indicated (Additional file 7, Figure S4A) suggesting that related bacteria might also exist in the salmon gut and could be the donor lineage of this gene and that this protein might play a role in host-animal interactions.

### Iron metabolism and the Fenton reaction

One of the most toxic byproducts of oxidative stress is hydroxyl anions generated via the Fenton reaction with oxygen and ferrous iron [92, 93]. Like other ROS, hydroxyl radicals can cause oxidative damage to DNA [94]. To prevent the Fenton reaction, organisms strive to keep free or labile iron levels low by storing iron in proteins such as Ferritin—a protein that can sequester thousands molecules of Fe [95]. *S. salmonicida* does not encode Ferritin, and a comparable Fe-sequestering protein has not yet been identified. In the absence of a *bona fide* Ferritin, it is unclear how *S. salmonicida* and other anaerobic eukaryotes lacking Ferritin shield their free iron from oxygen. We identified an array of genes encoding Fe-S and iron-containing proteins that were upregulated (Tables 3 and 4), including Ferritin-related RBR. These RBRs could serve as Fe storage systems in addition to performing their native function. It is also tempting to propose that the high number of cysteine-rich proteins predicted to localize throughout the cell [17] could also play a role in Fe-binding, since cysteine residues are often critical for metal binding. However, this hypothesis must be tested experimentally.

### Oxygen-induced pathogenicity

Within the animal gut, oxygen concentrations increase from the lumen to the epithelium [66], and thus organisms, such as *G. intestinalis* and *S. salmonicida*, that directly interact with the host are exposed to higher oxygen concentrations than when swimming in the lumen. Therefore, oxygen might serve as a signal for the induction of evasion- or invasion-related genes such as antigenic variation and secreted effectors proteins. In fact, similar aerotaxis models have been described for some bacterial pathogens [67].

To evade the host immune system, *G. intestinalis* uses cysteine-rich variable surface proteins (VSPs) [96]. Each individual *G. intestinalis* cell expresses only one VSP at the cell surface at a time by silencing all but one transcript using RNA interference [53, 97–99]. Every 6–13 divisions, the trophozoite switches which VSP is expressed by an incompletely understood mechanism. Previous reports have proposed that the cysteine-rich membrane proteins (CRMPs) of *S. salmonicida* might be analogous to these VSPs given their subcellular location to the outer membrane, including the flagella [17]. Interestingly, in the NAO cells, genes encoding cysteine-rich proteins were among the highest upregulated genes (Additional file 1; Fig. 3a). Future experimental work is necessary to determine if the *S. salmonicida* CRMP proteins contribute to antigenic variation. There are no homologues of these cysteine-rich proteins outside of diplomonads; however, similar massive gene family expansions have been observed in other parasitic eukaryotes. These include the leucine-rich BspA proteins of *Trichomonas vaginalis* [100] and *Entamoeba* [101], where, like the VSPs, these proteins might function in host interactions. In fact, the expression of the *bspA* genes might also be responsive to oxygen concentration as they were upregulated in *Trichomons vaginalis* transcriptome after oxidative stress [102].

In order to gain access to the blood, *S. salmonicida* likely disrupts the integrity of the epithelial cells by secreting proteases to degrade the tight junctions of the host. In other pathogens, secreted cysteine proteases weaken the host epithelium in order to traverse different mucosa [103, 104], destroying immune effectors [105] or in the case of *Entamoeba histolytica* degrade the host mucin. In fact, this cysteine protease is not found in avirulent species of *Entamoeba*, such as *E. dispar*, strongly suggesting this protease contributes to the invasive lifestyle of the parasite (reviewed in [106]). Indeed, genes encoding cysteine proteases were highly expressed in OXY and NAO cells suggesting these proteins might have similar roles in *S. salmonicida*. Intense proteolytic activity has been detected in the culture medium of other *Spironuclus* species [107]. Additional genes encoding proteins such as toxins, efflux pumps, and extracellular effector proteins were also found to be upregulated (Additional files 6 and 9); however, the lack of experimental evidence of these protein families makes it difficult to hypothesize their putative role during *S. salmonicida* infection. Future research should examine the role of the salmon gut microbiota on the oxidative stress response of *S. salmonicida* during infection as two recent studies of *E. histolytica* showed that *E. coli* can protect the amoeba from oxidative stress [108, 109].

### Evolutionary considerations

The invasive lifestyle of *S. salmonicida* is certainly the most distinguishing feature of this parasite compared to *G. intestinalis*. The molecular bases and origins of this capability remain elusive. Two evolutionary considerations for this fundamental difference are gene gain by lateral gene transfer (LGT) [41, 110] and gene family expansion [111, 112].

LGT has played an important role in the evolution of multiple lineages of eukaryotes [113] including anaerobic protists such as the gut pathogen *Blastocystis* [114] and free-living protists [115, 116]. Indeed, many *S. salmonicida* proteins related to adaptation to the host gut (e.g., efflux pumps, toxins, and secreted effector proteins) likely derive from LGT (Additional file 6). In one case, a protein was identified in *S. salmonicida* and no other eukaryote (secreted cysteine-rich protein; Additional file 6) and was likely transferred into the *S. salmonicida* genome via LGT. Many of these laterally acquired genes involved in both pathogenicity and oxygen tolerance were significantly upregulated in NAO or OXY cells. Such proteins are the prime candidates to investigate further as oxygen-responsive pathogenicity factors for invasive lifestyles.

When comparing the ROS response pathways of *G. intestinalis* and *S. salmonicida*, we observed that the *S. salmonicida* genes are often in multiple copies. This seems to be a genome-wide phenomenon whereby at least 324 orthologous groups have expanded in *S. salmonicida* compared to *G. intestinalis* (Additional file 11: Figure S7). This phenomenon has been observed in other parasites [111, 112]. Within these families, we sometimes observed differential regulation between gene copies (Additional file 1: Table S1) in the two conditions, suggesting environment-sensitive regulation of different gene copies. This correlates with the large number of transcription factor families found in *S. salmonicida* (Additional file 9; Additional file 10: Figure S6) that could allow for fine-tuned regulation of metabolism depending on the host environment.

Whether these laterally acquired genes or expanded gene families are essential for pathogenicity or simply interaction with other microbes in anaerobic environments is currently obscured due to the lack of knowledge on the basic biology of free-living protists from similar environments. Future studies examining the genomes of diverse free-living relatives of parasites will be essential to understand the molecular mechanism and evolution of parasitism.

## Conclusion

Here, we describe how the versatile metabolism, defense strategies, and pathogenicity factors of *S. salmonicida* respond to oxygen stress. These data provide the first molecular evidence for how this parasite can thrive across a spectrum of oxygen concentrations by directly detoxifying reactive oxygen species, increasing production of protective compounds such as cysteine or by sequestering ferrous iron. We further propose that *S. salmonicida* uses oxygen as a trigger for the induction of pathogenicity factors involved in immune evasion and invasion of the host epithelium. In particular, we suspect proteases and variable antigen presentation could play a key role in invading and evading the host. However, the lack of clear pathogenicity or invasion pathways typical of other parasites strongly suggests that *S. salmonicida* uses a unique mechanism of entering the host circulatory system. Future studies should interrogate the multitude of hypothetical proteins encoded by *S. salmonicida* that contain predicted secretion signals and predicted transmembrane domains and whose genes are upregulated in response to oxygen in order to illuminate the intriguing biology of this anaerobe.

## Materials and methods

### Cell culturing, RNA extraction, and sequencing

*Spironucleus salmonicida* (ATCC 50377) was maintained at 15 °C in modified liver digest, yeast extract, and iron (LYI) medium (pH 6.8) with limited headspace [15]. For each stress condition, four biological replicates were prepared. To expose the cultured cells to oxygen (OXY), cells were first grown to near-confluence (70–80% flask coverage) in T25 flasks filled with 55-mL media at which point, 30 mL of media was removed and cells were gently rocked for 1 h. To study the effects of antioxidants, the cells were grown in LYI media devoid of l-cysteine-HCl and ascorbic acid (no antioxidants; NAO) for 5 days. Removal of cysteine and ascorbic acid has previously been shown to induce oxidative damage and delay growth of *Giardia intestinalis* [54], and we therefore predicted a similar physiological response in *Spironucleus salmonicida*. To collect the control and oxygen-exposed cells, media were removed from the flask and 1 mL of Trizol (Life Technologies) was added to the cell monolayer and scraped with a cell scraper. The NAO-treated cells did not form a monolayer, and therefore, the cells were instead collected by centrifugation as previously described [15]. RNA was isolated by the Trizol method according to the manufacturer’s protocol. To eliminate any DNA that co-purified with the RNA, samples were treated with TURBO DNA-free procedure (Ambion) using the “rigorous protocol”. Poly(A) selection, sequencing library preparation (TruSeq), and sequencing (2 × 125 bp; Illumina HiSeq2500) were performed at the National Genomics Infrastructure at the Uppsala hub of SciLifeLab (Uppsala, Sweden). On average, 17.0 million read pairs were sequenced per sample (Additional file 1). Raw data is available on the European Nucleotide Archive accession number PRJEB29289.

### RNAseq analysis

The raw sequences were quality trimmed by Trimmomatic-0.36 with the following parameters: ILLUMINACLIP:TruSeq3-PE: 2:30:10:5:true, LEADING: 3, TRAILING: 3, SLIDINGWINDOW: 4:20, MINLEN: 50 [117] resulting in, on average, 16.2 million read pairs per sample (Additional file 1). Proficiency of read trimming was inspected manually using FASTQC [118]. Due to multiple identical loci within the genome, we aligned quality trimmed reads to an in silico transcriptome. Briefly, transcripts were generated by retrieving 150 bp upstream and downstream of the open reading frame for each gene model (GiardiaDB version 32), and redundant multi-copy genes in *S. salmonicida* that were more than 98% identical were collapsed with cd-hit (7802 total). [119]. Reads were mapped and quantified to this transcriptome using salmon with default parameters [120] with over 95% of reads (on average, 15.6 million read pairs) mapping per sample. Gene expression levels were assessed with the DESeq2 [121] using the “tximport” and “results” module (Additional file 12). Genes with adjusted *p* values (false discovery rates or *q* values) < 0.05 and absolute log2-fold changes (LFC) greater than 1.0 were considered differentially expressed. In general, we observed that *S. salmonicida* genes display a larger distribution of significantly differentially expressed genes than *G. intestinalis* [25]; therefore, we applied different absolute LFC cut-off of 0.7 for *G. intestinalis* to assess differential expression.

### Quantitative polymerase chain reaction

RNA was isolated as described above for four biological replicates in a separate growth experiment from the RNAseq samples. Total RNA was treated with DNAase as described above. Complementary DNA (cDNA) was synthesized using the RevertAid H Minus reverse transcriptase kit (Thermo) according to the manufacturer’s protocol using oligo dT primers and 10 ng of total RNA. Reactions devoid of reverse transcriptase were included in subsequent reactions as a “no RT negative control”. Primers were designed for six target genes (*rbr2*, SS50377_11802; *fld*, SS50377_13883; *nr*, SS50377_18652; *nadphor*, SS50377_19201; *msrA*, SS50377_17334; and *msrB*, SS50377_12928) and one reference gene (*fructokinase* SS50377_15261) (Additional file 1). All qPCRs were performed in triplicate using the Maxima SYBR Green/ROX qPCR master mix kit (Thermo) with 5 ng of template cDNA and 0.3 uM of each primer with the following cycling parameters: 95 °C, 15 min; [95 °C, 15 s; 60 °C, 30 s; 72 °C, 30 s] × 40 cycles and detected using Light Cycler 480 II (Roche). Primer efficiencies were tested using the same protocol using a dilution series (10, 2, 0.4, 0.08, 0.016, 0.0032 ng) of genomic DNA (Additional file 1). The quantification cycle (Cq) was averaged for the technical replicates. Relative quantification of cDNA derived from OXY- and NAO-treated cells was performed implementing primer efficiencies using the Pfaffl method comparing to the reference gene [122] and statistical analysis (Student’s *t* test of NAO or OXY versus the control cells) performed in Prism 8 (Additional file 4: Figure S2). Raw data and calculations are available [123].

### Phylogenetic methods, operon, and sequence analysis

All *S. salmonicida* gene copies for each protein of interest (NADHox, FDP, SOR, RBR, carotenoid isomerase, hemolysin (HL), MatE-type efflux pump, major facilitator superfamily protein (MFS), and bacterial-type cysteine-rich secretory protein (SCP)) were used as queries in BLAST [124] to retrieve the top 1000 hits against *nr*. Since we observed that many of the best hits were terrabacteria, we also retrieved the top 500 hits excluding terrabacteria. In cases where we retrieved other eukaryotic sequences, we would use these eukaryotic sequences as queries to retrieve an additional 500 hits. Prokaryotic and eukaryotic sequences greater than 60% or 80% similar respectively were removed using cd-hit [119]. We also surveyed genomic and transcriptomic data from free-living relatives of *S. salmonicida* described previously [125]. Sequences were aligned using mafft-linsi [126], and ambiguously aligned positions were removed using BMGE [127] with entropy (-h) scores greater than 0.7 under the BLOSUM30 substitution matrix. Initial phylogenies were made using FastTreeMP [128] under the LG model, and distantly related clades to the sequences of interest were removed. The final dataset was realigned as above and used for maximum-likelihood (ML) phylogenetic reconstruction using IQTREE [129] under the best fitting model using the Akaike information criterion corrected for number of parameters (AICc) assessed with IQTREE (-mset LG+C20,LG+C10,LG+C60,LG+C30,LG+C40,LG+C50 -cmax 7 -merit AICc). Ultrafast bootstrap (1000) were mapped on to the best scoring ML tree. For carotenoid isomerase, the 15 genes up- and downstream of the bacterial sequences most closely related to *Spironucleus* were manually interrogated for proteins related to electron transfer using an in-house python script. Output of these analyses is shown in Additional file 1. Alignments can be found on Figshare [130].

OrthoMCL information was retrieved from *Giardia*DB for *Giardia intestinalis* WB and *Spironucleus salmonicida*. In-house scripts were used to compare the number of gene copies per orthologue group in each organism. Subcellular localization prediction was performed using BUSCA (Bologna Unified Subcellular Component Annotator [131] using the “other eukaryotes” method) and DeepLoc (using the “BLOSUM” method [132]) webservers.

### Gene network and metabolic pathway analysis

All *S. salmonicida* gene annotations were retrieved from GiardiaDB (version 32) [17] and Uniprot. In-house gene lists from the *S. salmonicida* genome project were used to construct gene tables [17]. Fe-S clusters binding domains were predicted with Metal Predator [52]. Evolutionary gene network analysis was performed using EGN [133] and visualized with gephi [134]. Edge lengths and widths were weighted by pair-wise sequence identity. Transfection protocols and immunofluorescence methods are found in the supplementary material (Additional file 9).

## Additional files

Additional file 1: Read mapping statistics, gene expression information, and primer sequences. See file for details. (XLSX 2535 kb)

Additional file 2: **Figure S1.** Expression patterns of various *S. salmonicida* gene sets in OXY and NAO cells. (A) All *S. salmonicida* non-identical genes. *S. salmonicida* genes encoding hypothetical proteins with (B) homologues in other organisms, (C) a subset of (B) homologues found only *in S. salmonicida* and *G. intestinalis*, and (D) no homologues on the Genbank non-redundant database (2014). Each UpSetR graph represents the number of upregulated, downregulated, or not significant/no change (n.s./n.c.) with horizontal bars in each set as indicated. Points represent the comparison for each vertical bar with the total number of genes corresponding to each comparison are shown on top of the bar. For example, in panel A, there were 674 and 856 genes downregulated in OXY and NAO cells respectively, and 225 of these were downregulated in both conditions. (TIF 737 kb)

Additional file 3: Bioinformatic summary of protein domain architecture, predicted transmembrane domains and subcellular localization for the three sets of hypothetical proteins reported in Additional file 2: Figure S1. (XLSX 923 kb)

Additional file 4: **Figure S2.** Quantitative polymerase chain reaction of six target genes. For each gene, qPCRs were performed on four biological replicates each with three technical reaction replicates. Each dot represents the relative fold change of a gene within a biological replicate compared to an average of the control sample and was calculated using the Pfaffl method against the reference gene (*fructokinase*). Standard error bars are mapped onto the average of the four biological replicates. A Student’s *t* test was used to asses significant difference between the NAO (light grey) or OXY (dark grey) cells against the control cells (white). Significance values are as indicated: * < 0.01; ** < 0.001; *** < 0.0001. Raw data and calculations are deposited here [123]. (PDF 436 kb)

Additional file 5: **Figure S3.** Phylogenetic analysis suggests proteins related to oxygen stress response were acquired by lateral gene transfer. Phylogenetic trees were generated for (A) NADH oxidase, (B) Flavodiiron protein, (C) Superoxide reductase and (D) Rubrerythrin. Maximum likelihood (ML) phylogenies were generated using IQTREE under the indicated model of evolution. For visualization purposes, distantly related clades of prokaryotes were collapsed. Complete phylogenies can be found in Additional file 4 and [130]. Bipartition values from 1000 ultrafast bootstrap replicates were mapped onto the best scoring ML tree and labeled with a solid or open circle to represent bipartition values greater than 95 or 80 respectively. Bipartitions supporting diplomonad monophyly or sister relationship to prokaryotes are shown in grey circles and squares respectively. Genes that were significantly up or down regulated are indicated for OXY (left) and NAO (right) cells with up or down arrow respectively. Organisms are colored based on their taxonomic classification, eukaryotes (green), metamonads (light purple), fornicates (dark purple), archaea (orange) and bacteria (black). (TIF 4932 kb)

Additional file 6: Uncollapsed phylogenies of proteins related to oxidative stress response and host-pathogen interactions. For each gene, the total number of sites and taxa retained in the alignment and model of evolution used are indicated in the grey box. Bipartition support values from 1000 ultrafast bootstrap replicates were mapped onto the best scoring ML tree. Organisms are colored based on their taxonomic classification, eukaryotes (green), metamonads (light purple), fornicates (dark purple), archaea (orange) and bacteria (black). Gene expression pattern for each differentially expressed gene is shown with up or down arrows representing up and down-regulation in OXY (left) and NAO (right) cells. (PDF 273 kb)

Additional file 7: **Figure S4.** Phylogenetic analysis of carotenoid isomerase-like proteins and subcellular localization in *S. salmonicida*. (A) Tree was estimated using an alignment of 125 taxa and 414 sites using IQTREE under the LG+C20+F model of evolution. Bacterial, archaeal and eukaryotic sequences are shown in black, orange and purple respectively. The genomes of the closest prokaryotic relatives of the *S. salmonicida* sequences were manually investigated for genes related to oxygen defense in close proximity (i.e., within 15 genes) of the carotenoid isomerase gene indicated by closed black circles adjacent to each taxon. Branch supports are label with closed, open, or numbered for support values greater than 95, 80 and 50 respectively. PDI, protein disulfide isomerase; PRX, peroxiredoxin; FLD, flavodoxin (*distinct from *S. salmonicida* type); and NADHOx, NADH oxidase. (B) *S. salmonicida* cells transfected with a plasmid encoding the carotenoid isomerase gene (SS50377_15222) upstream of the OLLAS epitope tag. Antibodies raised against the OLLAS tag are stained in red while nuclei are stained in blue with DAPI. Scale bar, 20 μm. (PDF 1317 kb)

Additional file 8: **Figure S5.** Differential expression profiles of protease and the proteasome in OXY and NAO cells. (A) *S. salmonicida* proteases are colored based on their predicted subfamily type and plotted with respect to the LFC values. Only genes with |LFC| > 1 and adjusted *p*-values (FDR, padj) < 0.05 for OXY and NAO cells are shown. (B) Comparison of LFC values of genes encoding proteases across OXY (*x*-axis) and NAO (*y*-axis) cells where |LFC| > 1 and both (left panel) or either (right panel) padj conditions was less than 0.05. (C) Predicted proteasome of *S. salmonicida* reconstructed from the genome data. Genes are colored based on their differential expression value upregulated (red), downregulated(blue), and unchanged/insignificant (white). (PDF 5988 kb)

Additional file 9: Supplementary material – Supplementary methods and discussion of encystation, transcription factors and host-pathogen and pathogen-microbe interactions [135–148]. (PDF 52 kb)

Additional file 10: **Figure S6.** Expression patterns of *S. salmonicida* transcription factors in OXY and NAO cells. Evolutionary gene network of all differentially expressed transcription factors in OXY (purple) and/or NAO (orange) cells based on sequence similarity. Each node represents one gene encoding a cysteine-rich protein where edges were weighted by pair-wise sequence identity. Upregulated and downregulated genes are shown in red and blue respectively. Circle nodes with or without dots represent genes that were uniquely differentially regulated in OXY or NAO cells respectively; square nodes represent genes that were similarly differentially regulated in OXY and NAO cells. A summary of the gene expression changes for each condition are shown in the bottom right corner of the network with upregulated, downregulated and non-insignificant/no change (n.s./n.c) represented as up arrowheads, down arrowheads, or dashes respectively. (PDF 805 kb)

Additional file 11: **Figure S7.** The number of *G. intestinalis* orthologous groups with 1–5 gene copies (left) that are expanded in *S. salmonicida* (right). For example, 291 orthologous groups are encoded by only one gene in *G. intestinalis* (blue) and 179 of these orthologous groups have two gene copies in the *S. salmonicida* genome and the remainder have three or more copies. Only orthologous groups that had more gene copies in *S. salmonicida* than *G. intestinalis* are shown. Select examples of gene families discussed in this study are highlighted with their corresponding expression pattern in OXY and NAO cells respectively. Orthologous groups where at least one gene copy was upregulated in OXY or NAO cells are show with up arrowheads. (TIF 11060 kb)

Additional file 12: Differential expression workflow. R markdown file of data import and plot generation for DESeq2 analyses. *(PDF 61 kb)*
