# Supplementary figures and images for: Oxygen induces the expression of invasion and stress response genes in the anaerobic salmon parasite Spironucleus salmonicida
Source: BMC Biol. 2019 Mar 1;17:19. doi: 10.1186/s12915-019-0634-8 (PMC6397501; doi:10.1186/s12915-019-0634-8)

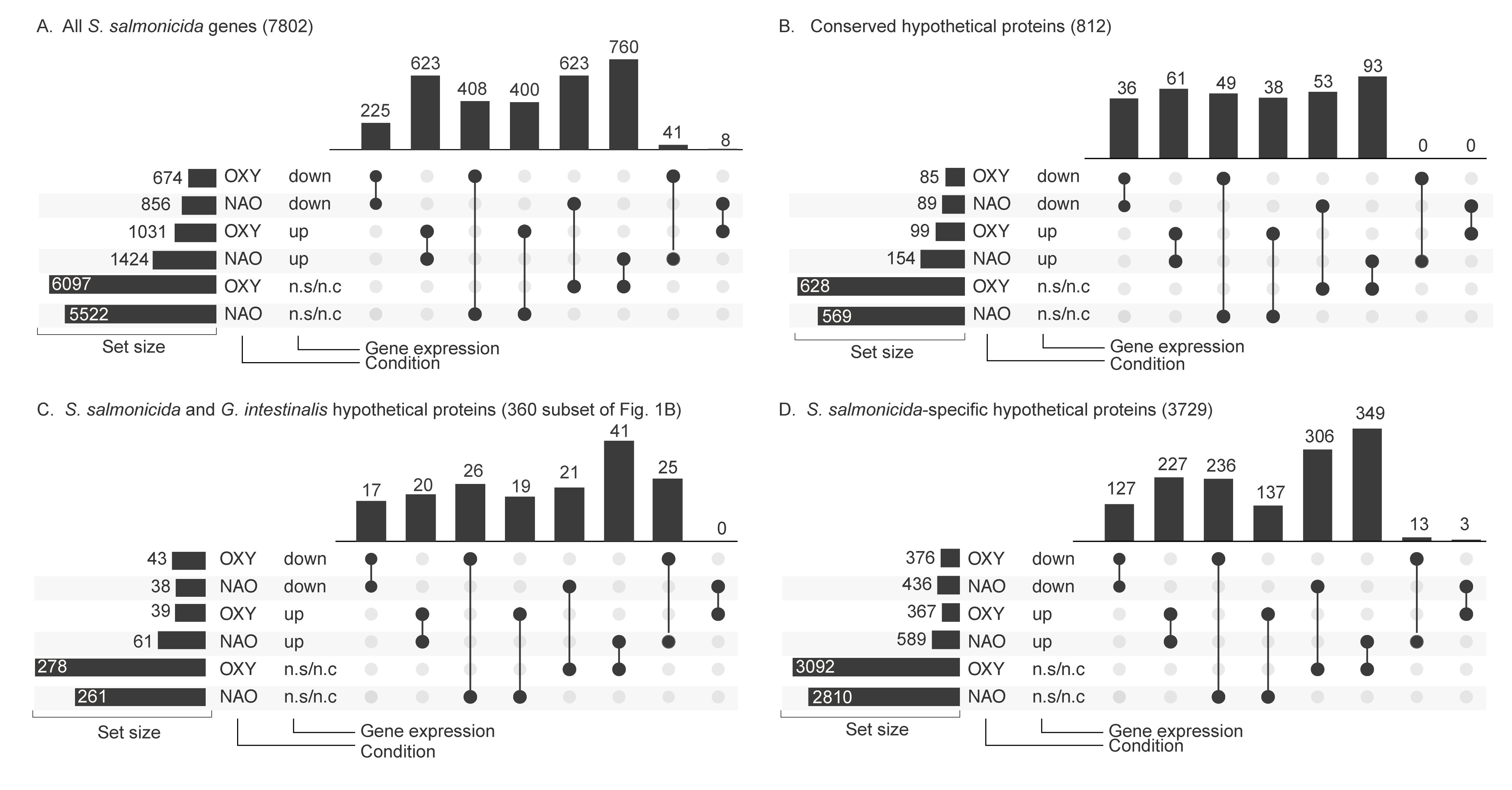

Supplement: Supplementary file 2 — Figure S1. Expression patterns of various S. salmonicida gene sets in OXY and NAO cells. (A) All S. salmonicida non-identical genes. S. salmonicida genes encoding hypothetical proteins with (B) homologues in other organisms, (C) a subset of (B) homologues found only in S. salmonicida and G. intestinalis, and (D) no homologues on the Genbank non-redundant database (2014). Each UpSetR graph represents the number of upregulated, downregulated, or not significant/no change (n.s./n.c.) with horizontal bars in each set as indicated. Points represent the comparison for each vertical bar with the total number of genes corresponding to each comparison are shown on top of the bar. For example, in panel A, there were 674 and 856 genes downregulated in OXY and NAO cells respectively, and 225 of these were downregulated in both conditions. (TIF 737 kb) [file 12915_2019_634_MOESM2_ESM.tif]

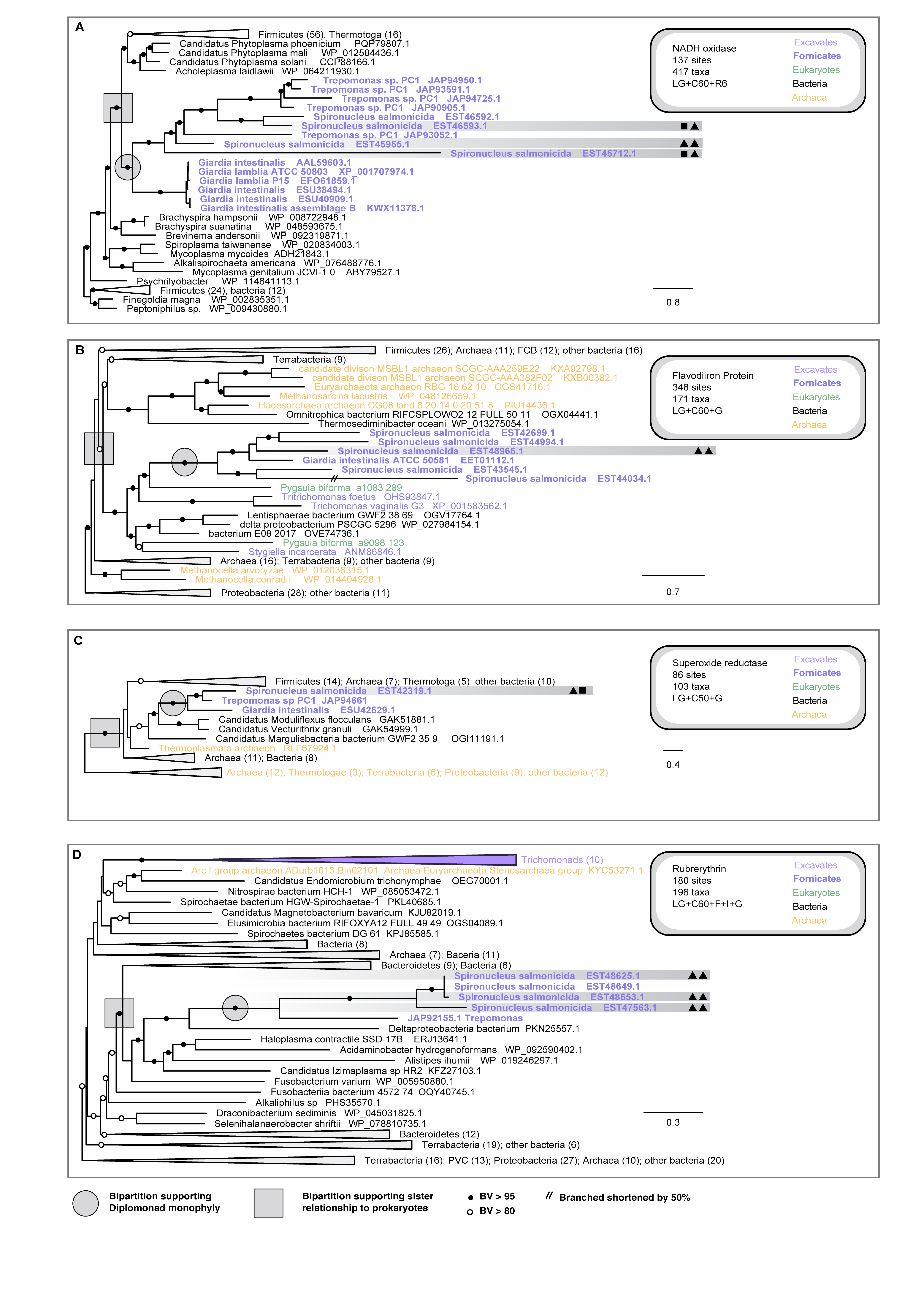

Supplement: Supplementary file 5 — Figure S3. Phylogenetic analysis suggests proteins related to oxygen stress response were acquired by lateral gene transfer. Phylogenetic trees were generated for (A) NADH oxidase, (B) Flavodiiron protein, (C) Superoxide reductase and (D) Rubrerythrin. Maximum likelihood (ML) phylogenies were generated using IQTREE under the indicated model of evolution. For visualization purposes, distantly related clades of prokaryotes were collapsed. Complete phylogenies can be found in Additional file 4 and [130]. Bipartition values from 1000 ultrafast bootstrap replicates were mapped onto the best scoring ML tree and labeled with a solid or open circle to represent bipartition values greater than 95 or 80 respectively. Bipartitions supporting diplomonad monophyly or sister relationship to prokaryotes are shown in grey circles and squares respectively. Genes that were significantly up or down regulated are indicated for OXY (left) and NAO (right) cells with up or down arrow respectively. Organisms are colored based on their taxonomic classification, eukaryotes (green), metamonads (light purple), fornicates (dark purple), archaea (orange) and bacteria (black). (TIF 4932 kb) [file 12915_2019_634_MOESM5_ESM.tif]

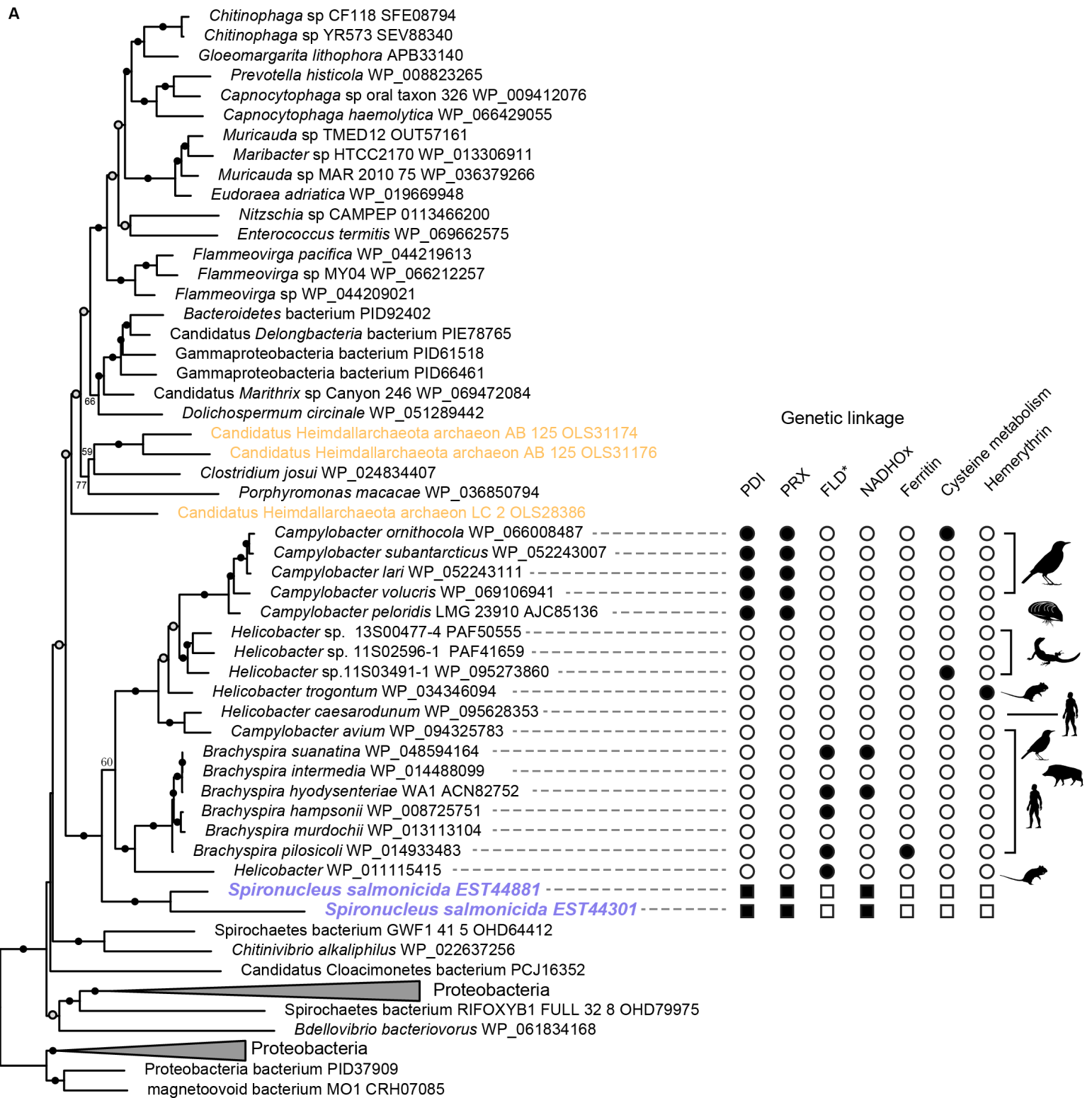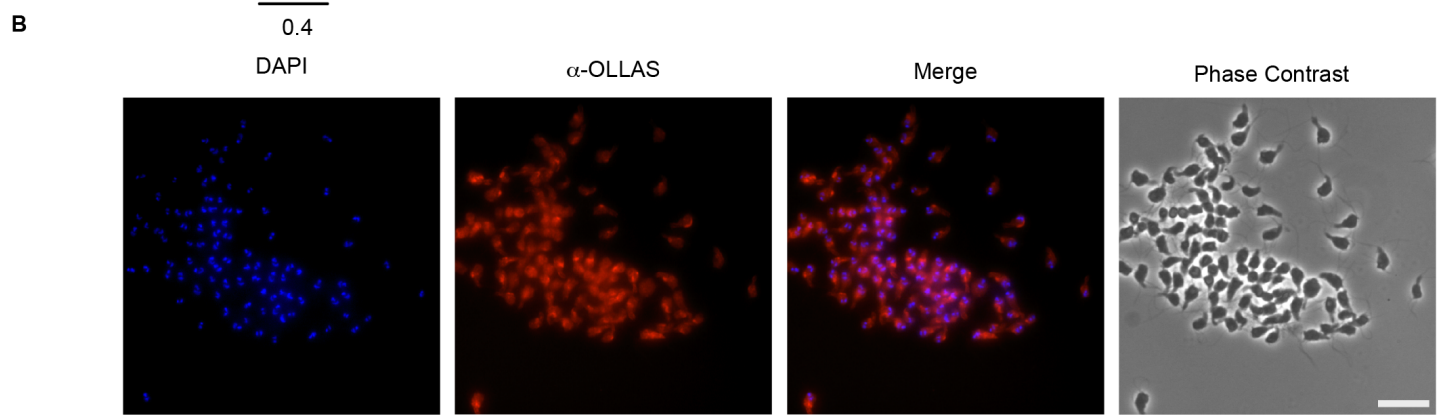

Supplement: Supplementary file 7 — Figure S4. Phylogenetic analysis of carotenoid isomerase-like proteins and subcellular localization in S. salmonicida. (A) Tree was estimated using an alignment of 125 taxa and 414 sites using IQTREE under the LG+C20+F model of evolution. Bacterial, archaeal and eukaryotic sequences are shown in black, orange and purple respectively. The genomes of the closest prokaryotic relatives of the S. salmonicida sequences were manually investigated for genes related to oxygen defense in close proximity (i.e., within 15 genes) of the carotenoid isomerase gene indicated by closed black circles adjacent to each taxon. Branch supports are label with closed, open, or numbered for support values greater than 95, 80 and 50 respectively. PDI, protein disulfide isomerase; PRX, peroxiredoxin; FLD, flavodoxin (*distinct from S. salmonicida type); and NADHOx, NADH oxidase. (B) S. salmonicida cells transfected with a plasmid encoding the carotenoid isomerase gene (SS50377_15222) upstream of the OLLAS epitope tag. Antibodies raised against the OLLAS tag are stained in red while nuclei are stained in blue with DAPI. Scale bar, 20 μm. (PDF 1317 kb) [file 12915_2019_634_MOESM7_ESM.pdf]

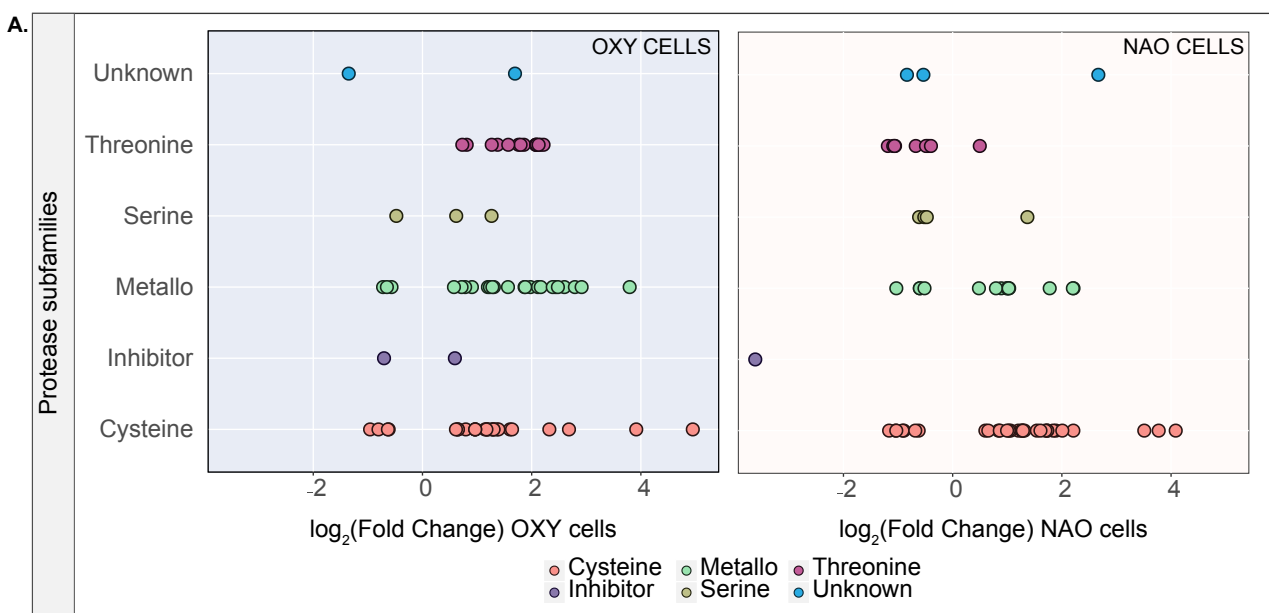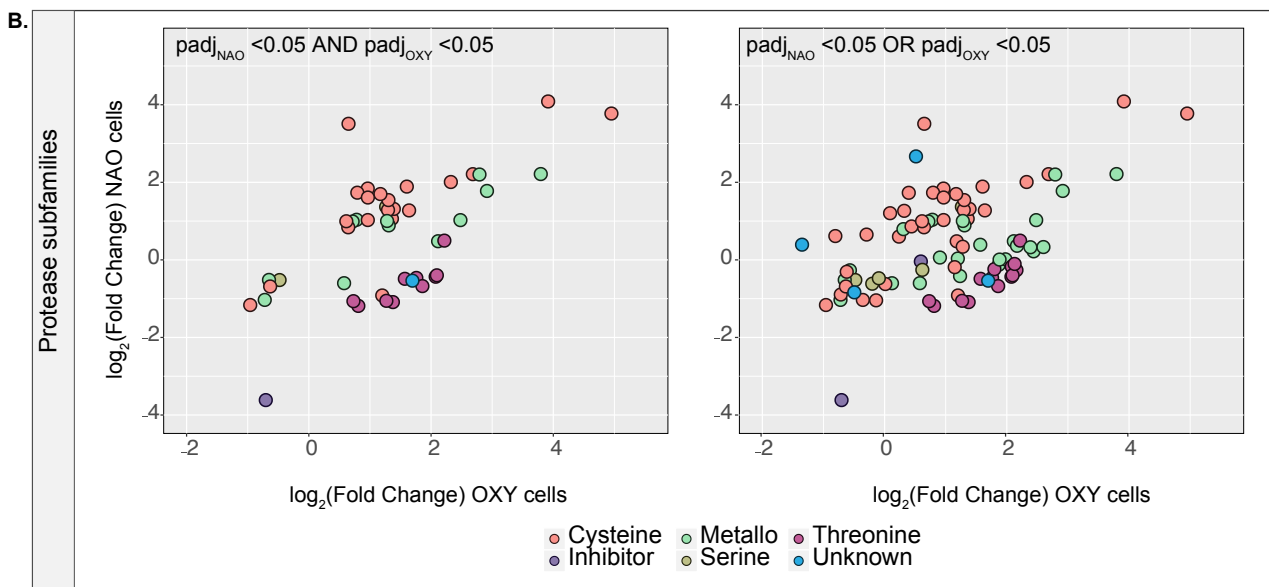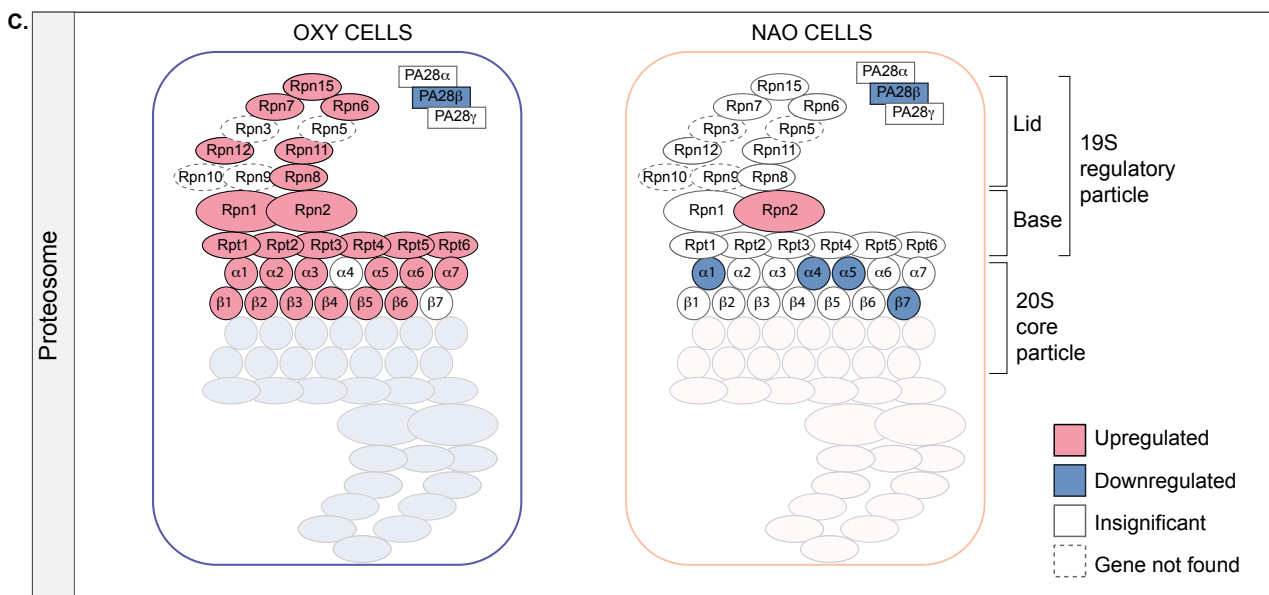

Supplement: Supplementary file 8 — Figure S5. Differential expression profiles of protease and the proteasome in OXY and NAO cells. (A) S. salmonicida proteases are colored based on their predicted subfamily type and plotted with respect to the LFC values. Only genes with |LFC| > 1 and adjusted p-values (FDR, padj) < 0.05 for OXY and NAO cells are shown. (B) Comparison of LFC values of genes encoding proteases across OXY (x-axis) and NAO (y-axis) cells where |LFC| > 1 and both (left panel) or either (right panel) padj conditions was less than 0.05. (C) Predicted proteasome of S. salmonicida reconstructed from the genome data. Genes are colored based on their differential expression value upregulated (red), downregulated(blue), and unchanged/insignificant (white). (PDF 5988 kb) [file 12915_2019_634_MOESM8_ESM.pdf]
