# Supplementary material for: Oxygen induces the expression of invasion and stress response genes in the anaerobic salmon parasite Spironucleus salmonicida
Source: BMC Biol. 2019 Mar 1;17:19. doi: 10.1186/s12915-019-0634-8 (PMC6397501; doi:10.1186/s12915-019-0634-8)
Supplement: Supplementary file 4 — Figure S2. Quantitative polymerase chain reaction of six target genes. For each gene, qPCRs were performed on four biological replicates each with three technical reaction replicates. Each dot represents the relative fold change of a gene within a biological replicate compared to an average of the control sample and was calculated using the Pfaffl method against the reference gene (fructokinase). Standard error bars are mapped onto the average of the four biological replicates. A Student’s t test was used to asses significant difference between the NAO (light grey) or OXY (dark grey) cells against the control cells (white). Significance values are as indicated: * < 0.01; ** < 0.001; *** < 0.0001. Raw data and calculations are deposited here [123]. (PDF 436 kb) [file 12915_2019_634_MOESM4_ESM.pdf]

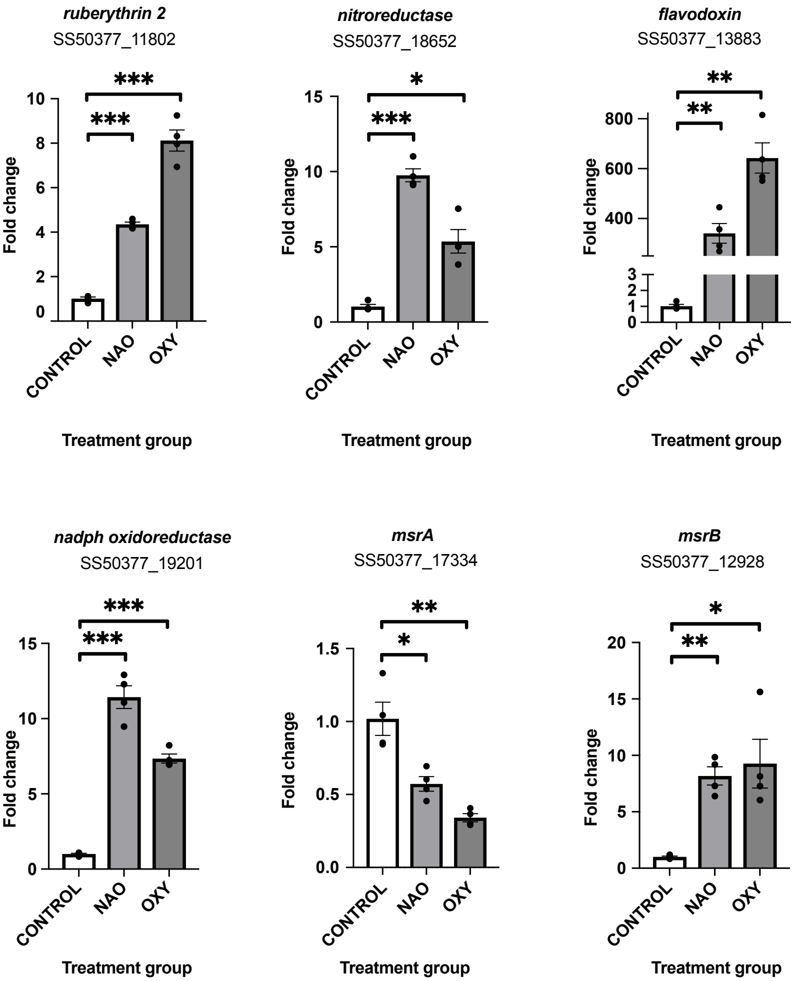
ruberythrin 2
SS50377_11802
***
***
Fold change
0
2
4
6
8
10
CONTROL
NAO
OXY
Treatment group
nitroreductase
SS50377_18652
*
***
Fold change
0
5
10
15
CONTROL
NAO
OXY
Treatment group
flavodoxin
SS50377_13883
**
**
Fold change
0
1
2
3
400
600
800
CONTROL
NAO
OXY
Treatment group
nadph oxidoreductase
SS50377_19201
***
***
Fold change
0
5
10
15
CONTROL
NAO
OXY
Treatment group
msrA
SS50377_17334
**
*
Fold change
0.0
0.5
1.0
1.5
CONTROL
NAO
OXY
Treatment group
msrB
SS50377_12928
*
**
Fold change
0
5
10
15
20
CONTROL
NAO
OXY
Treatment group
