# Supplementary material for: Oxygen induces the expression of invasion and stress response genes in the anaerobic salmon parasite Spironucleus salmonicida
Source: BMC Biol. 2019 Mar 1;17:19. doi: 10.1186/s12915-019-0634-8 (PMC6397501; doi:10.1186/s12915-019-0634-8)
Supplement: Supplementary file 6 — Uncollapsed phylogenies of proteins related to oxidative stress response and host-pathogen interactions. For each gene, the total number of sites and taxa retained in the alignment and model of evolution used are indicated in the grey box. Bipartition support values from 1000 ultrafast bootstrap replicates were mapped onto the best scoring ML tree. Organisms are colored based on their taxonomic classification, eukaryotes (green), metamonads (light purple), fornicates (dark purple), archaea (orange) and bacteria (black). Gene expression pattern for each differentially expressed gene is shown with up or down arrows representing up and down-regulation in OXY (left) and NAO (right) cells. (PDF 273 kb) [file 12915_2019_634_MOESM6_ESM.pdf]

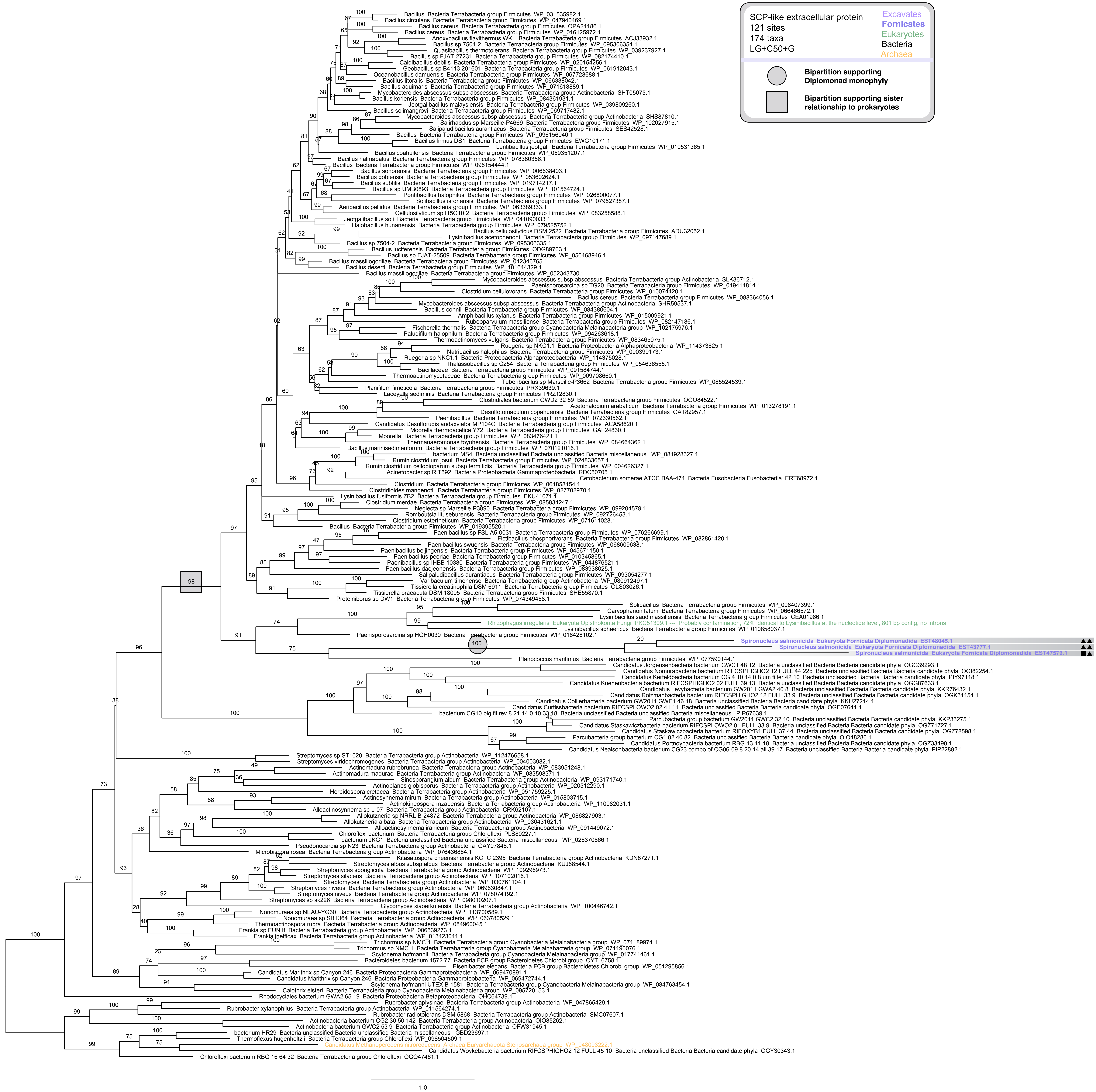
SCP-like extracellular protein
121 sites
174 taxa
LG+C50+G
Excavates
Fornicates
Eukaryotes
Bacteria
Archaea
Bipartition supporting Diplomonad monophyly
Bipartition supporting sister relationship to prokaryotes
Spironucleus salmonicida Eukaryota Fornicata Diplomonadida EST48045.1
Spironucleus salmonicida Eukaryota Fornicata Diplomonadida EST43777.1
Spironucleus salmonicida Eukaryota Fornicata Diplomonadida EST47579.1
Rhizophagus irregularis Eukaryota Opisthokonta Fungi PKC51309.1 ← Probably contamination, 72% identical to Lysinibacillus at the nucleotide level, 801 bp contig, no introns
Candidatus Methanoperedens nitroreducens Archaea Euryarchaeota Stenosarchaea group WP_048093222.1
1.0

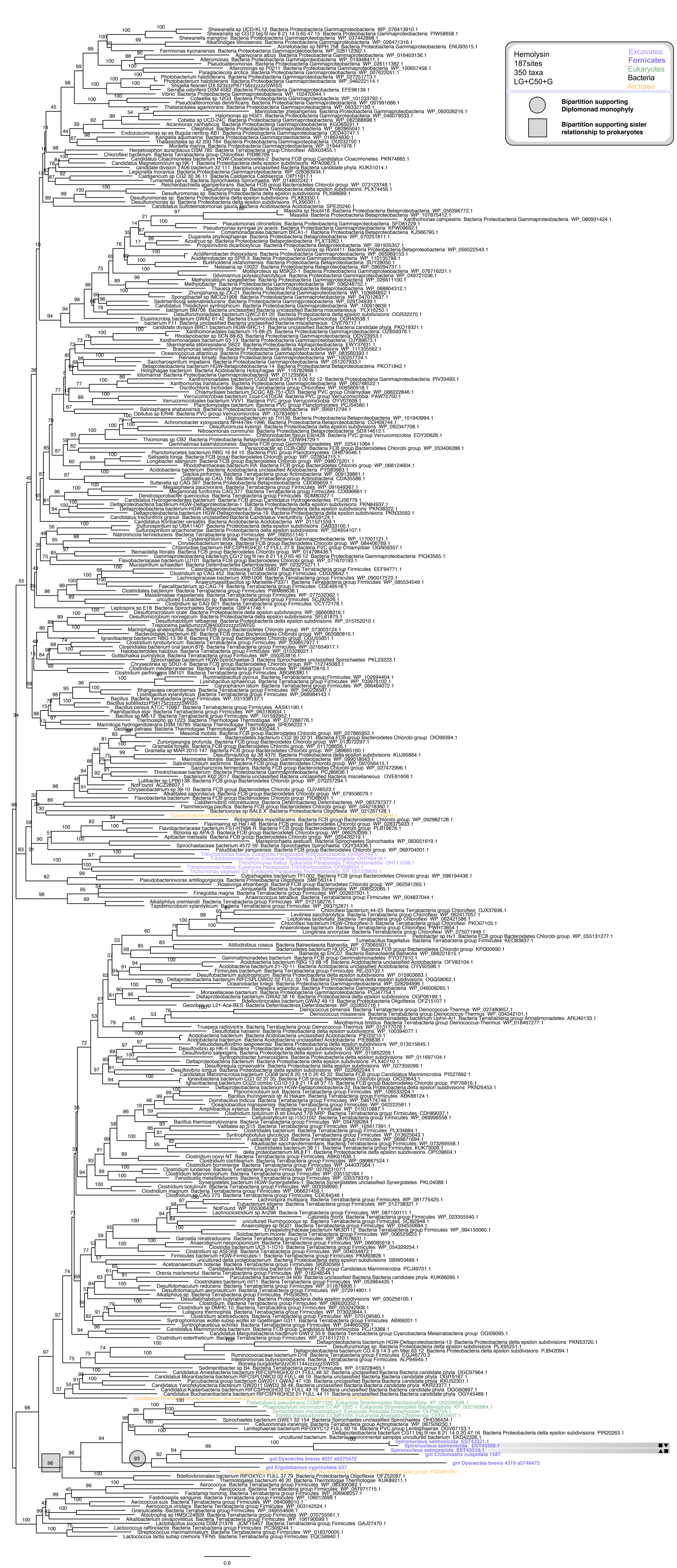
Hemolysin
187sites
350 taxa
LG+C50+G
Excavates
Fornicates
Eukaryotes
Bacteria
Archaea
Bipartition supporting Diplomonad monophyly
Bipartition supporting sister relationship to prokaryotes
gnl Dysnectes brevis 4037 a5275472
gnl Dysnectes brevis 4319 a5748473
0.6

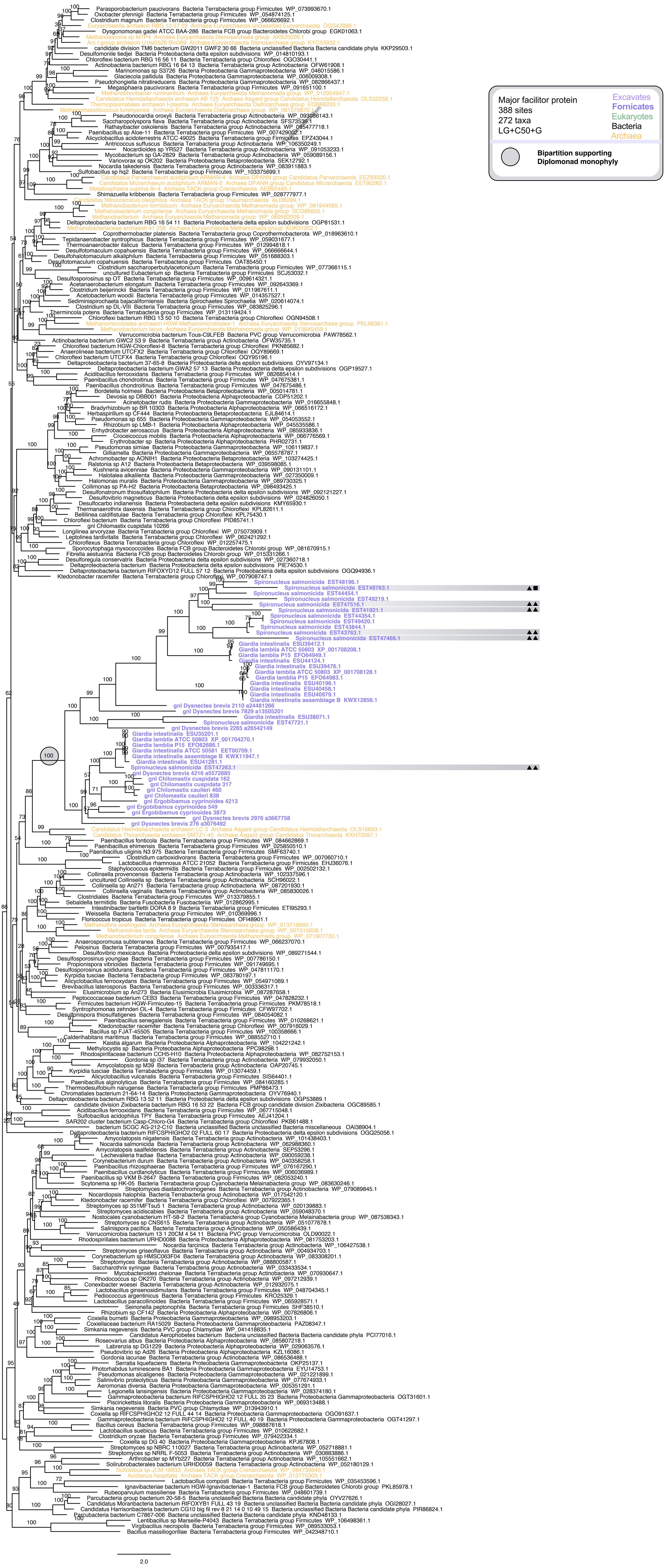
Major facilitor protein
388 sites
272 taxa
LG+C50+G
Excavates
Fornicates
Eukaryotes
Bacteria
Archaea
Bipartition supporting
Diplomonad monophyly
2.0

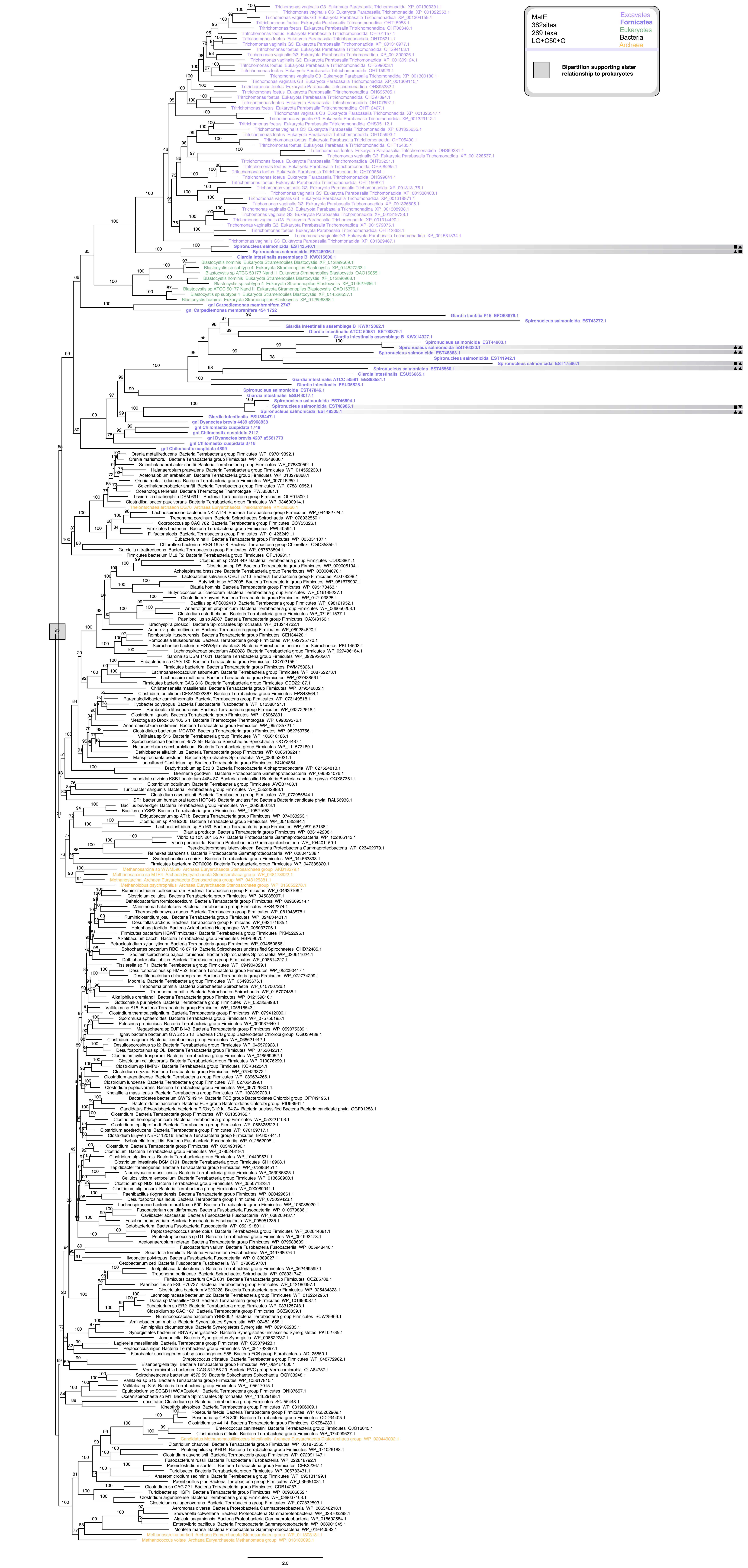
MatE
382sites
289 taxa
LG+C50+G
Excavates
Fornicates
Eukaryotes
Bacteria
Archaea
Bipartition supporting sister relationship to prokaryotes
2.0

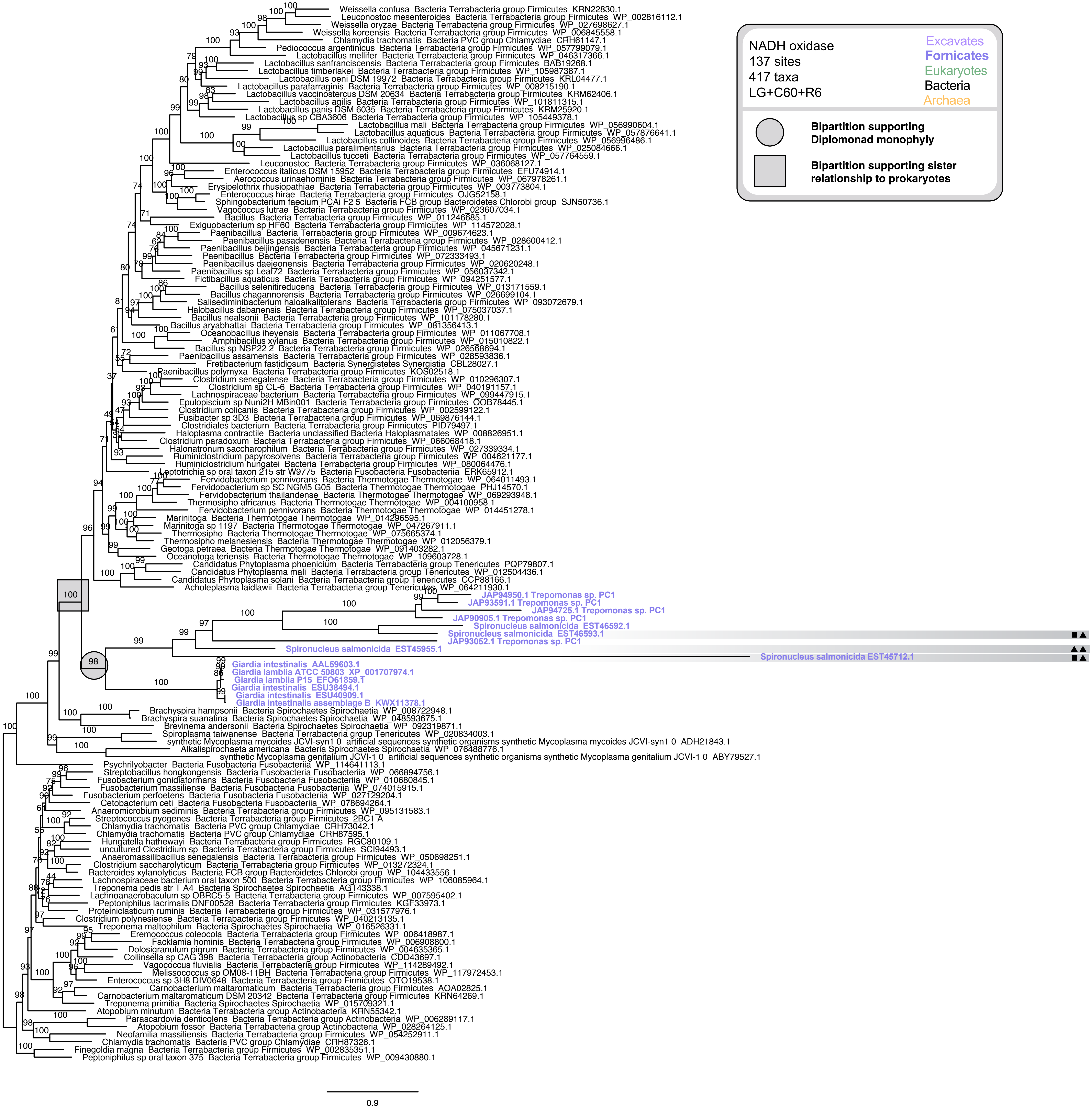
NADH oxidase
137 sites
417 taxa
LG+C60+R6
Excavates
Fornicates
Eukaryotes
Bacteria
Archaea
Bipartition supporting Diplomonad monophyly
Bipartition supporting sister relationship to prokaryotes
Weissella confusa Bacteria Terrabacteria group Firmicutes KRN22830.1
Leuconostoc mesenteroides Bacteria Terrabacteria group Firmicutes WP_002816112.1
Weissella oryzae Bacteria Terrabacteria group Firmicutes WP_027698627.1
Weissella koreensis Bacteria Terrabacteria group Firmicutes WP_006845558.1
Chlamydia trachomatis Bacteria PVC group Chlamydiae CRH61147.1
Pediococcus argentinicus Bacteria Terrabacteria group Firmicutes WP_057799079.1
Lactobacillus mellifer Bacteria Terrabacteria group Firmicutes WP_046317366.1
Lactobacillus sanfranciscensis Bacteria Terrabacteria group Firmicutes BAB19268.1
Lactobacillus timberlakei Bacteria Terrabacteria group Firmicutes WP_105987387.1
Lactobacillus oeni DSM 19972 Bacteria Terrabacteria group Firmicutes KRL04477.1
Lactobacillus parafarraginis Bacteria Terrabacteria group Firmicutes WP_008215190.1
Lactobacillus vaccinostercus DSM 20634 Bacteria Terrabacteria group Firmicutes KRM62406.1
Lactobacillus agilis Bacteria Terrabacteria group Firmicutes WP_101811315.1
Lactobacillus panis DSM 6035 Bacteria Terrabacteria group Firmicutes KRM25920.1
Lactobacillus sp CBA3606 Bacteria Terrabacteria group Firmicutes WP_105449378.1
Lactobacillus mali Bacteria Terrabacteria group Firmicutes WP_056990604.1
Lactobacillus aquaticus Bacteria Terrabacteria group Firmicutes WP_057876641.1
Lactobacillus collinoides Bacteria Terrabacteria group Firmicutes WP_056996486.1
Lactobacillus paralimentarius Bacteria Terrabacteria group Firmicutes WP_025084666.1
Lactobacillus tucceti Bacteria Terrabacteria group Firmicutes WP_057764559.1
Leuconostoc Bacteria Terrabacteria group Firmicutes WP_036068127.1
Enterococcus italicus DSM 15952 Bacteria Terrabacteria group Firmicutes EFU74914.1
Aerococcus urinaehominis Bacteria Terrabacteria group Firmicutes WP_067978261.1
Erysipelothrix rhusiopathiae Bacteria Terrabacteria group Firmicutes WP_003773804.1
Enterococcus hirae Bacteria Terrabacteria group Firmicutes OJG52158.1
Sphingobacterium faecium PCAi F2 5 Bacteria FCB group Bacteroidetes Chlorobi group SJN50736.1
Vagococcus lutrae Bacteria Terrabacteria group Firmicutes WP_023607034.1
Bacillus Bacteria Terrabacteria group Firmicutes WP_011246685.1
Exiguobacterium sp HF60 Bacteria Terrabacteria group Firmicutes WP_114572028.1
Paenibacillus Bacteria Terrabacteria group Firmicutes WP_009674623.1
Paenibacillus pasadenensis Bacteria Terrabacteria group Firmicutes WP_028600412.1
Paenibacillus beijingensis Bacteria Terrabacteria group Firmicutes WP_045671231.1
Paenibacillus Bacteria Terrabacteria group Firmicutes WP_072333493.1
Paenibacillus daejeonensis Bacteria Terrabacteria group Firmicutes WP_020620248.1
Paenibacillus sp Leaf72 Bacteria Terrabacteria group Firmicutes WP_056037342.1
Fictibacillus aquaticus Bacteria Terrabacteria group Firmicutes WP_094251577.1
Bacillus selenitireducens Bacteria Terrabacteria group Firmicutes WP_013171559.1
Bacillus chagannorensis Bacteria Terrabacteria group Firmicutes WP_026699104.1
Salisediminibacterium haloalkalitolerans Bacteria Terrabacteria group Firmicutes WP_093072679.1
Halobacillus dabanensis Bacteria Terrabacteria group Firmicutes WP_075037037.1
Bacillus nealsonii Bacteria Terrabacteria group Firmicutes WP_101178280.1
Bacillus aryabhattai Bacteria Terrabacteria group Firmicutes WP_081356413.1
Oceanobacillus iheyensis Bacteria Terrabacteria group Firmicutes WP_011067708.1
Amphibacillus xylanus Bacteria Terrabacteria group Firmicutes WP_015010822.1
Bacillus sp NSP22 2 Bacteria Terrabacteria group Firmicutes WP_026568694.1
Paenibacillus assamensis Bacteria Terrabacteria group Firmicutes WP_028593836.1
Fretibacterium fastidiosum Bacteria Synergistetes Synergistia CBL28027.1
Paenibacillus polymyxa Bacteria Terrabacteria group Firmicutes KOS02518.1
Clostridium senegalense Bacteria Terrabacteria group Firmicutes WP_010296307.1
Clostridium sp CL-6 Bacteria Terrabacteria group Firmicutes WP_040191157.1
Lachnospiraceae bacterium Bacteria Terrabacteria group Firmicutes WP_099447915.1
Epulopiscium sp Nuni2H MBin001 Bacteria Terrabacteria group Firmicutes OOB78445.1
Clostridium colicanis Bacteria Terrabacteria group Firmicutes WP_002599122.1
Fusibacter sp 3D3 Bacteria Terrabacteria group Firmicutes WP_069876144.1
Clostridiales bacterium Bacteria Terrabacteria group Firmicutes PID79497.1
Haloplasma contractile Bacteria unclassified Bacteria Haloplasmatales WP_008826951.1
Clostridium paradoxum Bacteria Terrabacteria group Firmicutes WP_066068418.1
Halonatronum saccharophilum Bacteria Terrabacteria group Firmicutes WP_027339334.1
Ruminiclostridium papyrosolvens Bacteria Terrabacteria group Firmicutes WP_004621177.1
Ruminiclostridium hungatei Bacteria Terrabacteria group Firmicutes WP_080064476.1
Leptotrichia sp oral taxon 215 str W9775 Bacteria Fusobacteria Fusobacteriia ERK65912.1
Fervidobacterium pennivorans Bacteria Thermotogae Thermotogae WP_064011493.1
Fervidobacterium sp SC NGM5 G05 Bacteria Thermotogae Thermotogae PHJ14570.1
Fervidobacterium thailandense Bacteria Thermotogae Thermotogae WP_069293948.1
Thermosipho africanus Bacteria Thermotogae Thermotogae WP_004100958.1
Fervidobacterium pennivorans Bacteria Thermotogae Thermotogae WP_014451278.1
Marinitoga Bacteria Thermotogae Thermotogae WP_014296595.1
Marinitoga sp 1197 Bacteria Thermotogae Thermotogae WP_047267911.1
Thermosipho Bacteria Thermotogae Thermotogae WP_075665374.1
Thermosipho melanesiensis Bacteria Thermotogae Thermotogae WP_012056379.1
Geotoga petraea Bacteria Thermotogae Thermotogae WP_091403282.1
Oceanotoga teriensis Bacteria Thermotogae Thermotogae WP_109603728.1
Candidatus Phytoplasma phoenicium Bacteria Terrabacteria group Tenericutes PQP79807.1
Candidatus Phytoplasma mali Bacteria Terrabacteria group Tenericutes WP_012504436.1
Candidatus Phytoplasma solani Bacteria Terrabacteria group Tenericutes CCP88166.1
Acholeplasma laidlawii Bacteria Terrabacteria group Tenericutes WP_064211930.1
JAP94950.1 Trepomonas sp. PC1
JAP93591.1 Trepomonas sp. PC1
JAP94725.1 Trepomonas sp. PC1
JAP90905.1 Trepomonas sp. PC1
Spironucleus salmonicida EST46592.1
Spironucleus salmonicida EST46593.1
JAP93052.1 Trepomonas sp. PC1
Spironucleus salmonicida EST45955.1
Spironucleus salmonicida EST45712.1
Giardia intestinalis AAL59603.1
Giardia lamblia ATCC 50803 XP_001707974.1
Giardia lamblia P15 EFO61859.1
Giardia intestinalis ESU38494.1
Giardia intestinalis ESU40909.1
Giardia intestinalis assemblage B KWX11378.1
Brachyspira hampsonii Bacteria Spirochaetes Spirochaetia WP_008722948.1
Brachyspira suanatina Bacteria Spirochaetes Spirochaetia WP_048593675.1
Brevinema andersonii Bacteria Spirochaetes Spirochaetia WP_092319871.1
Spiroplasma taiwanense Bacteria Terrabacteria group Tenericutes WP_020834003.1
synthetic Mycoplasma mycoides JCVI-syn1 0 artificial sequences synthetic organisms synthetic Mycoplasma mycoides JCVI-syn1 0 ADH21843.1
Alkalispirochaeta americana Bacteria Spirochaetes Spirochaetia WP_076488776.1
synthetic Mycoplasma genitalium JCVI-1 0 artificial sequences synthetic organisms synthetic Mycoplasma genitalium JCVI-1 0 ABY79527.1
Psychrilyobacter Bacteria Fusobacteria Fusobacteriia WP_114641113.1
Streptobacillus hongkongensis Bacteria Fusobacteria Fusobacteriia WP_066894756.1
Fusobacterium gonidiaformans Bacteria Fusobacteria Fusobacteriia WP_010680845.1
Fusobacterium massiliense Bacteria Fusobacteria Fusobacteriia WP_074015915.1
Fusobacterium perfoetens Bacteria Fusobacteria Fusobacteriia WP_027129204.1
Cetobacterium ceti Bacteria Fusobacteria Fusobacteriia WP_078694264.1
Anaeromicrobium sediminis Bacteria Terrabacteria group Firmicutes WP_095131583.1
Streptococcus pyogenes Bacteria Terrabacteria group Firmicutes 2BC1 A
Chlamydia trachomatis Bacteria PVC group Chlamydiae CRH73042.1
Chlamydia trachomatis Bacteria PVC group Chlamydiae CRH87595.1
Hungatella hathewayi Bacteria Terrabacteria group Firmicutes RGC80109.1
uncultured Clostridium sp Bacteria Terrabacteria group Firmicutes SCI94493.1
Anaeromassilibacillus senegalensis Bacteria Terrabacteria group Firmicutes WP_050698251.1
Clostridium saccharolyticum Bacteria Terrabacteria group Firmicutes WP_013272324.1
Bacteroides xylanolyticus Bacteria FCB group Bacteroidetes Chlorobi group WP_104433556.1
Lachnospiraceae bacterium oral taxon 500 Bacteria Terrabacteria group Firmicutes WP_106085964.1
Treponema pedis str T A4 Bacteria Spirochaetes Spirochaetia AGT43338.1
Lachnoanaerobaculum sp OBRC5-5 Bacteria Terrabacteria group Firmicutes WP_007595402.1
Peptoniphilus lacrimalis DNF00528 Bacteria Terrabacteria group Firmicutes KGF33973.1
Proteiniclasticum ruminis Bacteria Terrabacteria group Firmicutes WP_031577976.1
Clostridium polynesiense Bacteria Terrabacteria group Firmicutes WP_040213135.1
Treponema maltophilum Bacteria Spirochaetes Spirochaetia WP_016526331.1
Eremococcus coleocola Bacteria Terrabacteria group Firmicutes WP_006418987.1
Facklamia hominis Bacteria Terrabacteria group Firmicutes WP_006908800.1
Dolosigranulum pigrum Bacteria Terrabacteria group Firmicutes WP_004635365.1
Collinsella sp CAG 398 Bacteria Terrabacteria group Actinobacteria CDD43697.1
Vagococcus fluvialis Bacteria Terrabacteria group Firmicutes WP_114289492.1
Melissococcus sp OM08-11BH Bacteria Terrabacteria group Firmicutes WP_117972453.1
Enterococcus sp 3H8 DIV0648 Bacteria Terrabacteria group Firmicutes OTO19538.1
Carnobacterium maltaromaticum Bacteria Terrabacteria group Firmicutes AOA02825.1
Carnobacterium maltaromaticum DSM 20342 Bacteria Terrabacteria group Firmicutes KRN64269.1
Treponema primitia Bacteria Spirochaetes Spirochaetia WP_015709321.1
Atopobium minutum Bacteria Terrabacteria group Actinobacteria KRN55342.1
Parascardovia denticolens Bacteria Terrabacteria group Actinobacteria WP_006289117.1
Atopobium fossor Bacteria Terrabacteria group Actinobacteria WP_028264125.1
Neofamilia massiliensis Bacteria Terrabacteria group Firmicutes WP_054252911.1
Chlamydia trachomatis Bacteria PVC group Chlamydiae CRH87326.1
Finegoldia magna Bacteria Terrabacteria group Firmicutes WP_002835351.1
Peptoniphilus sp oral taxon 375 Bacteria Terrabacteria group Firmicutes WP_009430880.1
0.9

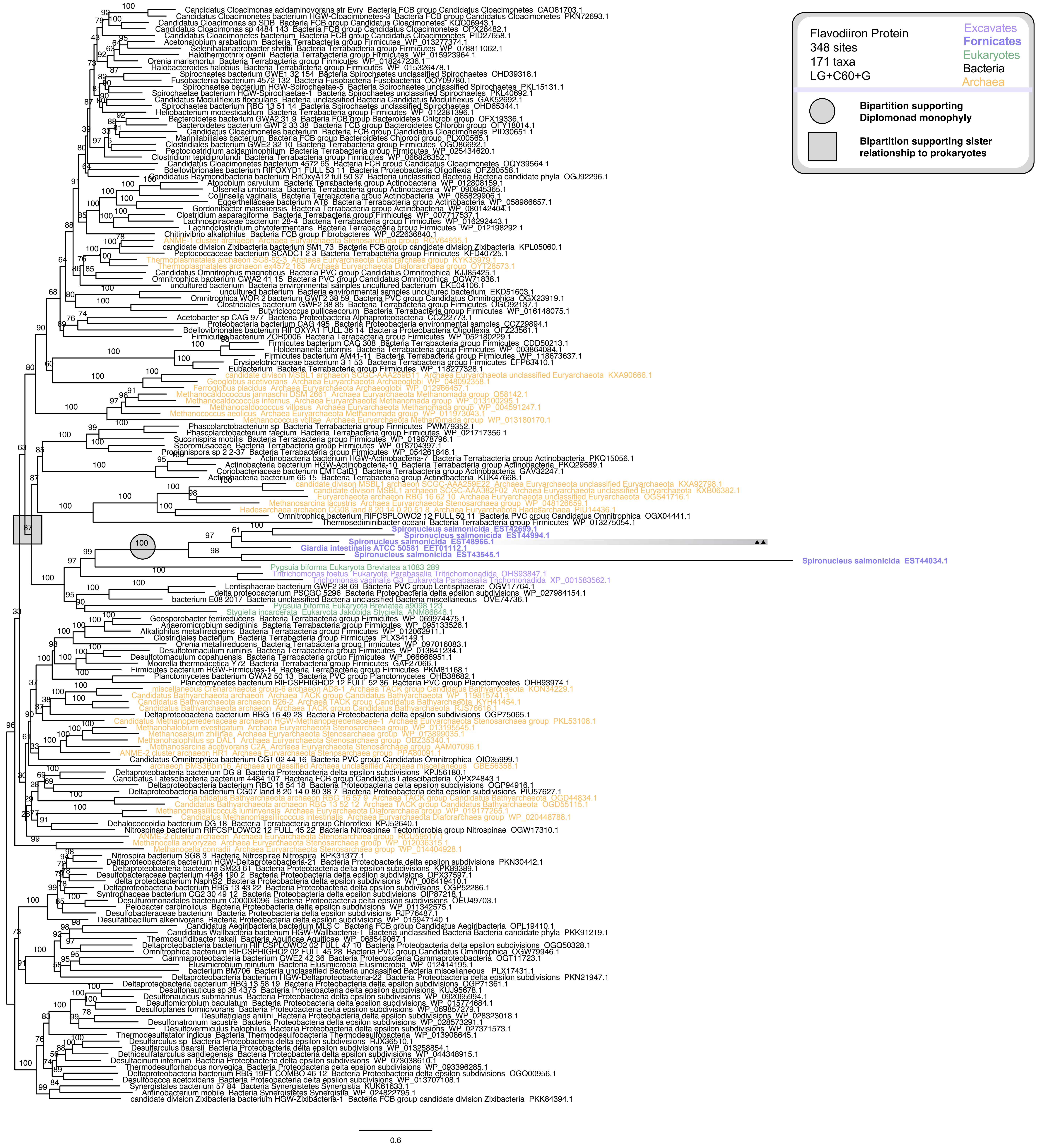

Flavodiiron Protein
348 sites
171 taxa
LG+C60+G
Excavates
Fornicates
Eukaryotes
Bacteria
Archaea
Bipartition supporting Diplomonad monophyly
Bipartition supporting sister relationship to prokaryotes
0.6

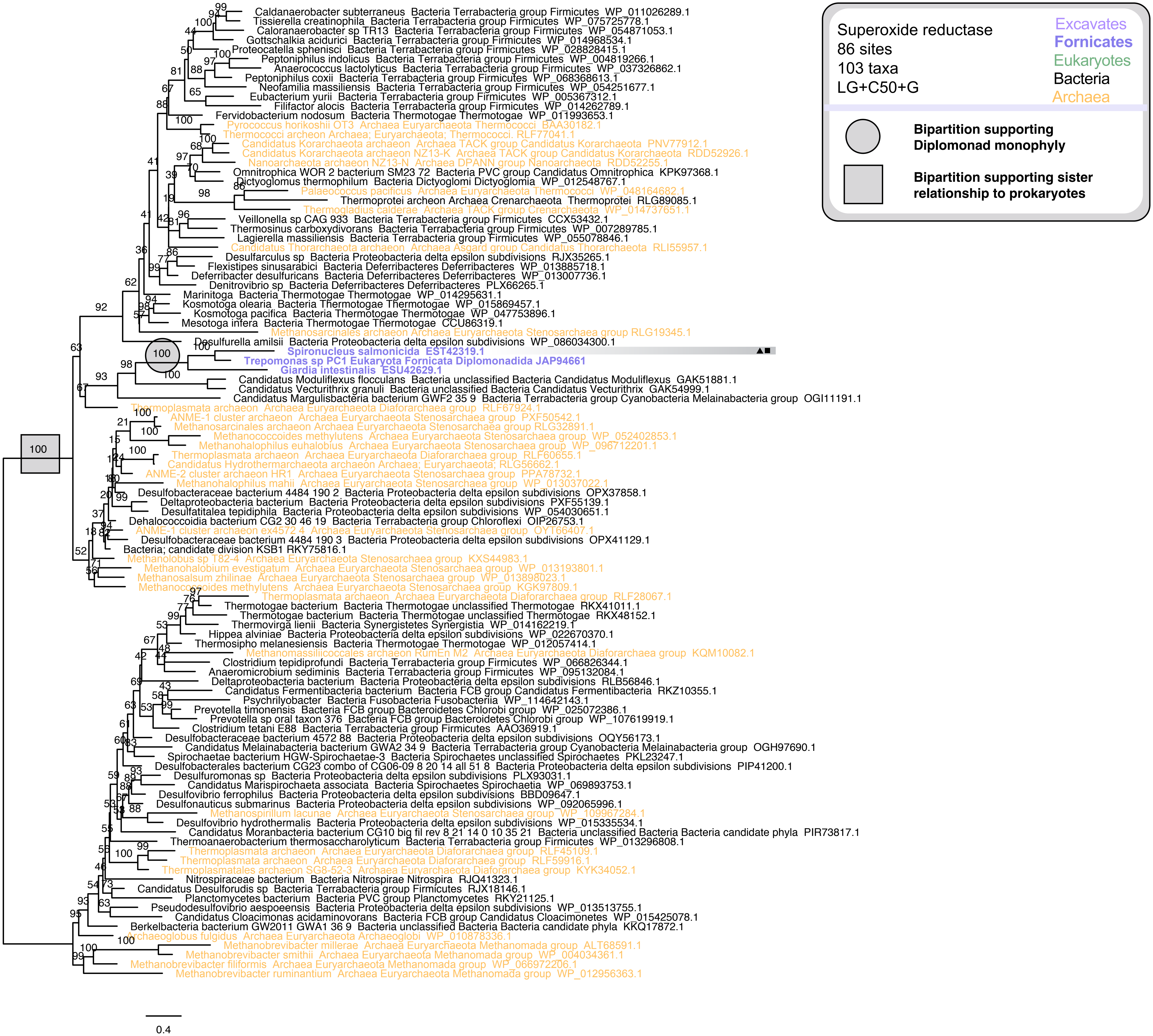
Superoxide reductase
86 sites
103 taxa
LG+C50+G
Excavates
Fornicates
Eukaryotes
Bacteria
Archaea
Bipartition supporting Diplomonad monophyly
Bipartition supporting sister relationship to prokaryotes
Caldanaerobacter subterraneus Bacteria Terrabacteria group Firmicutes WP_011026289.1
Tissierella creatinophila Bacteria Terrabacteria group Firmicutes WP_075725778.1
Caloranaerobacter sp TR13 Bacteria Terrabacteria group Firmicutes WP_054871053.1
Gottschalkia acidurici Bacteria Terrabacteria group Firmicutes WP_014968534.1
Proteocatella sphenisci Bacteria Terrabacteria group Firmicutes WP_028828415.1
Peptoniphilus indolicus Bacteria Terrabacteria group Firmicutes WP_004819266.1
Anaerococcus lactolyticus Bacteria Terrabacteria group Firmicutes WP_037326862.1
Peptoniphilus coxii Bacteria Terrabacteria group Firmicutes WP_068368613.1
Neofamilia massiliensis Bacteria Terrabacteria group Firmicutes WP_054251677.1
Eubacterium yurii Bacteria Terrabacteria group Firmicutes WP_005367312.1
Filifactor alocis Bacteria Terrabacteria group Firmicutes WP_014262789.1
Fervidobacterium nodosum Bacteria Thermotogae Thermotogae WP_011993653.1
Pyrococcus horikoshii OT3 Archaea Euryarchaeota Thermococci BAA30182.1
Thermococci archeon Archaea; Euryarchaeota; Thermococci. RLF77041.1
Candidatus Korarchaeota archaeon Archaea TACK group Candidatus Korarchaeota PNV77912.1
Candidatus Korarchaeota archaeon NZ13-K Archaea TACK group Candidatus Korarchaeota RDD52926.1
Nanoarchaeota archaeon NZ13-N Archaea DPANN group Nanoarchaeota RDD52255.1
Omnitrophica WOR 2 bacterium SM23 72 Bacteria PVC group Candidatus Omnitrophica KPK97368.1
Dictyoglomus thermophilum Bacteria Dictyoglomi Dictyoglomia WP_012548767.1
Palaeococcus pacificus Archaea Euryarchaeota Thermococci WP_048164682.1
Thermoprotei archeon Archaea Crenarchaeota Thermoprotei RLG89085.1
Thermogladius calderae Archaea TACK group Crenarchaeota WP_014737651.1
Veillonella sp CAG 933 Bacteria Terrabacteria group Firmicutes CCX53432.1
Thermosinus carboxydivorans Bacteria Terrabacteria group Firmicutes WP_007289785.1
Lagierella massiliensis Bacteria Terrabacteria group Firmicutes WP_055078846.1
Candidatus Thorarchaeota archaeon Archaea Asgard group Candidatus Thorarchaeota RLI55957.1
Desulfarculus sp Bacteria Proteobacteria delta epsilon subdivisions RJX35265.1
Flexistipes sinusarabici Bacteria Deferribacteres Deferribacteres WP_013885718.1
Deferribacter desulfuricans Bacteria Deferribacteres Deferribacteres WP_013007736.1
Denitrovibrio sp Bacteria Deferribacteres Deferribacteres PLX66265.1
Marinitoga Bacteria Thermotogae Thermotogae WP_014295631.1
Kosmotoga olearia Bacteria Thermotogae Thermotogae WP_015869457.1
Kosmotoga pacifica Bacteria Thermotogae Thermotogae WP_047753896.1
Mesotoga infera Bacteria Thermotogae Thermotogae CCU86319.1
Methanosarcinales archaeon Archaea Euryarchaeota Stenosarchaea group RLG19345.1
Desulfurella amilsii Bacteria Proteobacteria delta epsilon subdivisions WP_086034300.1
Spironucleus salmonicida EST42319.1
Trepomonas sp PC1 Eukaryota Fornicata Diplomonadida JAP94661
Giardia intestinalis ESU42629.1
Candidatus Moduliflexus flocculans Bacteria unclassified Bacteria Candidatus Moduliflexus GAK51881.1
Candidatus Vecturithrix granuli Bacteria unclassified Bacteria Candidatus Vecturithrix GAK54999.1
Candidatus Margulisbacteria bacterium GWF2 35 9 Bacteria Terrabacteria group Cyanobacteria Melainabacteria group OGI11191.1
Thermoplasmata archaeon Archaea Euryarchaeota Diaforarchaea group RLF67924.1
ANME-1 cluster archaeon Archaea Euryarchaeota Stenosarchaea group PXF50542.1
Methanosarcinales archaeon Archaea Euryarchaeota Stenosarchaea group RLG32891.1
Methanococcoides methylutens Archaea Euryarchaeota Stenosarchaea group WP_052402853.1
Methanohalophilus euhalobius Archaea Euryarchaeota Stenosarchaea group WP_096712201.1
Thermoplasmata archaeon Archaea Euryarchaeota Diaforarchaea group RLF60655.1
Candidatus Hydrothermarchaeota archaeon Archaea; Euryarchaeota; RLG56662.1
ANME-2 cluster archaeon HR1 Archaea Euryarchaeota Stenosarchaea group PPA78732.1
Methanohalophilus mahii Archaea Euryarchaeota Stenosarchaea group WP_013037022.1
Desulfobacteraceae bacterium 4484 190 2 Bacteria Proteobacteria delta epsilon subdivisions OPX37858.1
Deltaproteobacteria bacterium Bacteria Proteobacteria delta epsilon subdivisions PXF55139.1
Desulfatitalea tepidiphila Bacteria Proteobacteria delta epsilon subdivisions WP_054030651.1
Dehalococcoidia bacterium CG2 30 46 19 Bacteria Terrabacteria group Chloroflexi OIP26753.1
ANME-1 cluster archaeon ex4572 4 Archaea Euryarchaeota Stenosarchaea group OYT66407.1
Desulfobacteraceae bacterium 4484 190 3 Bacteria Proteobacteria delta epsilon subdivisions OPX41129.1
Bacteria; candidate division KSB1 RKY75816.1
Methanolobus sp T82-4 Archaea Euryarchaeota Stenosarchaea group KXS44983.1
Methanohalobium evestigatum Archaea Euryarchaeota Stenosarchaea group WP_013193801.1
Methanosalsum zhilinae Archaea Euryarchaeota Stenosarchaea group WP_013898023.1
Methanococcoides methylutens Archaea Euryarchaeota Stenosarchaea group KGK97809.1
Thermoplasmata archaeon Archaea Euryarchaeota Diaforarchaea group RLF28067.1
Thermotogae bacterium Bacteria Thermotogae unclassified Thermotogae RKX41011.1
Thermotogae bacterium Bacteria Thermotogae unclassified Thermotogae RKX48152.1
Thermovirga lienii Bacteria Synergistetes Synergistia WP_014162219.1
Hippea alviniae Bacteria Proteobacteria delta epsilon subdivisions WP_022670370.1
Thermosipho melanesiensis Bacteria Thermotogae Thermotogae WP_012057414.1
Methanomassiliicoccales archaeon RumEn M2 Archaea Euryarchaeota Diaforarchaea group KQM10082.1
Clostridium tepidiprofundi Bacteria Terrabacteria group Firmicutes WP_066826344.1
Anaeromicrobium sediminis Bacteria Terrabacteria group Firmicutes WP_095132084.1
Deltaproteobacteria bacterium Bacteria Proteobacteria delta epsilon subdivisions RLB56846.1
Candidatus Fermentibacteria bacterium Bacteria FCB group Candidatus Fermentibacteria RKZ10355.1
Psychrilyobacter Bacteria Fusobacteria Fusobacteriia WP_114642143.1
Prevotella timonensis Bacteria FCB group Bacteroidetes Chlorobi group WP_025072386.1
Prevotella sp oral taxon 376 Bacteria FCB group Bacteroidetes Chlorobi group WP_107619919.1
Clostridium tetani E88 Bacteria Terrabacteria group Firmicutes AAO36919.1
Desulfobacteraceae bacterium 4572 88 Bacteria Proteobacteria delta epsilon subdivisions OQY56173.1
Candidatus Melainabacteria bacterium GWA2 34 9 Bacteria Terrabacteria group Cyanobacteria Melainabacteria group OGH97690.1
Spirochaetae bacterium HGW-Spirochaetae-3 Bacteria Spirochaetes unclassified Spirochaetes PKL23247.1
Desulfobacterales bacterium CG23 combo of CG06-09 8 20 14 all 51 8 Bacteria Proteobacteria delta epsilon subdivisions PIP41200.1
Desulfuromonas sp Bacteria Proteobacteria delta epsilon subdivisions PLX93031.1
Candidatus Marispirochaeta associata Bacteria Spirochaetes Spirochaetia WP_069893753.1
Desulfovibrio ferrophilus Bacteria Proteobacteria delta epsilon subdivisions BBD09647.1
Desulfonauticus submarinus Bacteria Proteobacteria delta epsilon subdivisions WP_092065996.1
Methanospirillum lacunae Archaea Euryarchaeota Stenosarchaea group WP_109967284.1
Desulfovibrio hydrothermalis Bacteria Proteobacteria delta epsilon subdivisions WP_015335534.1
Candidatus Moranbacteria bacterium CG10 big fil rev 8 21 14 0 10 35 21 Bacteria unclassified Bacteria Bacteria candidate phyla PIR73817.1
Thermoanaerobacterium thermosaccharolyticum Bacteria Terrabacteria group Firmicutes WP_013296808.1
Thermoplasmata archaeon Archaea Euryarchaeota Diaforarchaea group RLF45109.1
Thermoplasmata archaeon Archaea Euryarchaeota Diaforarchaea group RLF59916.1
Thermoplasmatales archaeon SG8-52-3 Archaea Euryarchaeota Diaforarchaea group KYK34052.1
Nitrospiraceae bacterium Bacteria Nitrospirae Nitrospira RJQ41323.1
Candidatus Desulforudis sp Bacteria Terrabacteria group Firmicutes RJX18146.1
Planctomycetes bacterium Bacteria PVC group Planctomycetes RKY21125.1
Pseudodesulfovibrio aespoeensis Bacteria Proteobacteria delta epsilon subdivisions WP_013513755.1
Candidatus Cloacimonas acidaminovorans Bacteria FCB group Candidatus Cloacimonetes WP_015425078.1
Berkelbacteria bacterium GW2011 GWA1 36 9 Bacteria unclassified Bacteria Bacteria candidate phyla KKQ17872.1
Archaeoglobus fulgidus Archaea Euryarchaeota Archaeoglobi WP_010878336.1
Methanobrevibacter millerae Archaea Euryarchaeota Methanomada group ALT68591.1
Methanobrevibacter smithii Archaea Euryarchaeota Methanomada group WP_004034361.1
Methanobrevibacter filiformis Archaea Euryarchaeota Methanomada group WP_066972206.1
Methanobrevibacter ruminantium Archaea Euryarchaeota Methanomada group WP_012956363.1
0.4

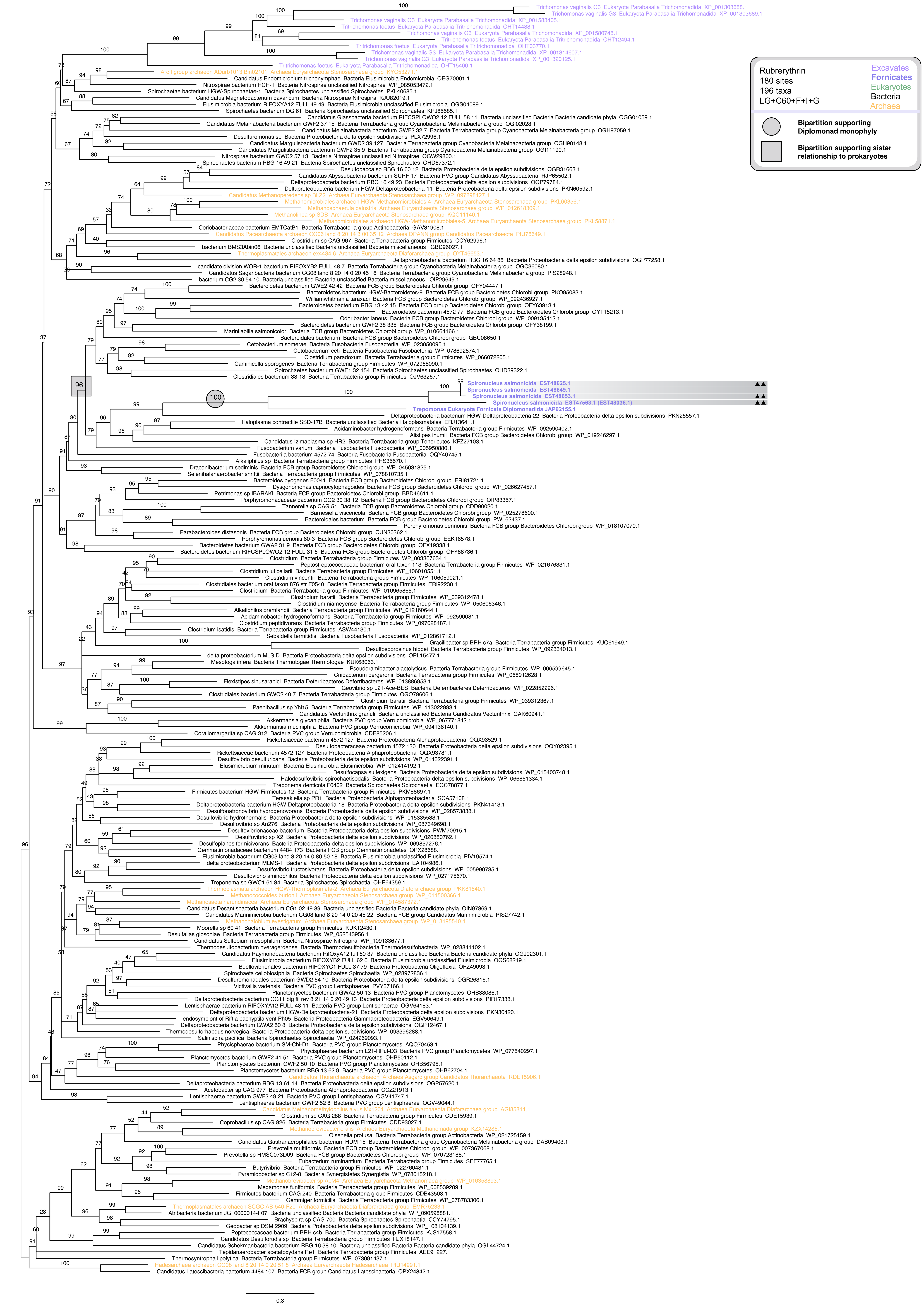

Rubrerythrin
180 sites
196 taxa
LG+C60+F+I+G
Excavates
Fornicates
Eukaryotes
Bacteria
Archaea
Bipartition supporting Diplomonad monophyly
Bipartition supporting sister relationship to prokaryotes
0.3
