# Supplementary material for: Oxygen induces the expression of invasion and stress response genes in the anaerobic salmon parasite Spironucleus salmonicida
Source: BMC Biol. 2019 Mar 1;17:19. doi: 10.1186/s12915-019-0634-8 (PMC6397501; doi:10.1186/s12915-019-0634-8)
Supplement: Supplementary file 9 — Supplementary material – Supplementary methods and discussion of encystation, transcription factors and host-pathogen and pathogen-microbe interactions [135–148]. (PDF 52 kb) [file 12915_2019_634_MOESM9_ESM.pdf]

# SUPPLEMENTARY RESULTS & DISCUSSION

## *Encystation*

The life cycle of *S. salmonicida* and whether there is a cyst life stage remains elusive. Cysts have been observed in other species of *Spironucleus* [5,13] that infect mice and birds, however there have been no convincing molecular evidence for cyst formation in fish parasites. The encystation process in *G. intestinalis* has been well studied [2,3]. *Giardia intestinalis* cyst walls are composed of three cyst wall proteins (CWP1-3) and the amino sugar N-acetylgalactosamine (GalNAc) [135–137]. Previous studies identified genes encoding eight cyst wall proteins 1 (CWP1; corresponding to five unique gene sequences), three CWP2, and GalNAc biosynthesis proteins encoded in the *S. salmonicida* genome [17]. One of these genes (SS50377_15904) was heterologously expressed in *G. intestinalis* and shown to localize to the cyst wall and encystation specific vesicles during encystation [17] suggesting *S. salmonicida* might have a cyst stage. In the oxygen-treated cells, none of these genes were differentially expressed. However, in the NAO condition we observed that one *cwp1* gene (SS50377_16858) and all three *cwp2* genes (SS50377_16197, SS50377_18399, SS50377_14854, SS50377_12130) were upregulated, albeit with relatively low (i.e., less than 100) recruited reads (Additional File 1).

The other component of the *Giardia intestinalis* cyst wall, GalNAc, is synthesized from the glycolytic intermediate fructose-6-phosphate by five different enzymes (glucosamine 6-phosphate deaminase, glucosamine 6-phophate N-acetylase, phosphoacetyl glucoseamine mutase, UDP-N-acetylglucosamine pyrophosphorylase, and UDP-N-acetylglucosamine 4-epimerase). In OXY and NAO cells, two genes encoding glucosamine 6-phosphate deaminase were upregulated (SS50377_11665 and SS50377_15226 respectively) and in NAO cells, the gene encoding the final synthesis of GalNAc (UDP-N-acetylglucosamine 4-epimerase; SS50377_17322) was downregulated. However, the remaining genes display no significant change in gene expression (Additional File 1).

## *Transcription factors*

We identified 134 transcription factors in the *Spironucleus salmonicida* genome, many of which are members of the Myb-like family [17]. We performed evolutionary genetic network analysis on all transcription factors and shaded the major transcription factor families based on

the annotation (Additional File 10: Figure S6). In general, most transcription factors were not significantly differentially regulated. In OXY and NAO cells, we observed 16 and 23 upregulated and 20 and 11 downregulated transcription factors, respectively (Additional File 10: Figure S6). Interestingly, only 10 transcription factors showed similarly differential expression patterns in both OXY and NAO cells (squares, Additional File 10: Figure S6). This suggests that these two oxygen stressors each induce their own transcriptional response.

### *Orthologue analysis*

To further understand the differences between *G. intestinalis* and *S. salmonicida* genomes, we examined the number of genes found in each orthologous group shared between the two organisms. We were most interested in the number of orthologous groups that had an increase in gene copy number in *S. salmonicida* compared to *G. intestinalis* (Additional File 1, Additional File 11, Figure S7). A total of 342 orthologues groups were found to have an increase in the number of gene copies in *S. salmonicida* compared to *G. intestinalis*. Some of these orthologuous groups include proteins already discussed above (e.g., Flavodoxin, Myb and proteases, Additional File 11 Figure S7). The most drastic gene family expansions were seen where *G. intestinalis* encodes one (Myb) or three (Cathepsin) copies of these proteins and *Spironucleus* encodes 28 and 12, respectively.

### *Host:pathogen and microbiota:pathogen interaction*

During gut infection, *Spironucleus* has to interacts with and defend itself against host cells and other members of the host's microbiota. Some gut microbes routinely use secreted proteins or use efflux pumps to modulate their environment. *Entamoeba histolytica* is known to perturb the composition of the host microbiome [138] potentially by degrading antimicrobial peptides or by preferential digestion of certain prokaryotic species (reviewed in [106]). We examined the expression pattern and evolutionary history genes encoding the following putative effector proteins: bacterial-type cysteine-rich secretory protein (SCP family), the pore-forming toxin hemolysin III and two efflux pumps.

Three genes encoding a putative bacterial-type cysteine-rich secretory protein (SCP family; TIGR02909) were found in the genome; SS50377_11811 and SS50377_16518 were upregulated in OXY and NAO cells while SS50377_12270 was only upregulated in NAO cells. These proteins are a subset of the CAP protein family (PF00188) consisting of cysteine-rich secretory proteins, antigen 5, and pathogenesis-related 1 proteins (reviewed in [139]). The SCP

proteins in particular are mostly found in endospore forming bacteria including Bacilliales (YkwD in *B. subtilis*) and might function as a serine protease [140], however we could not find any reliable reports of this function. In some fungi, CAP proteins have been linked to pathogenicity [141–143] although, these sequences are quite distinct from those we identified in *S. salmonicida* (i.e., they are not able to be retrieved with BLASTP). While the *S. salmonicida* CAP proteins are not predicted to contain a signal peptide by SignalP, they do have a predicted transmembrane domain and are predicted to function in the endomembrane system (by BUSCA analysis) or other membranous compartment (DeepLoc). We suspect that the divergent nature of the signal peptides [17] obscures their prediction with bioinformatic tools. To investigate the evolutionary history and subfamily classification of these genes, we performed a phylogenetic analysis of the *S. salmonicida* genes with their closest homologues (Additional File 6). We were unable to identify any other eukaryotic homologues of this particular subfamily of SCPs. We found that the *S. salmonicida* genes branch together with maximum support in a large clade of firmicutes (BV=98) to the exclusion of a clade of candidate phyla radiation bacteria (BV=96). This strongly suggests that these genes were acquired by *S. salmonicida* via lateral gene transfer. However, we could not predict the function of these protein as there are no closely related characterized representatives.

We also identified three genes encoding hemolysin III in the *S. salmonicida* genome, one of which (SS50377_16993) was upregulated in OXY cells. These proteins have been linked to hemolytic activity in *Bacillus cereus* [144]. Phylogenetic analysis reveals that Fornicates (e.g., *Spironucleus, Chilomastix, Dysnectes* and *Ergobibamus* species) form a robustly supported clade (BV=93) (Additional File 6). Interestingly, some stramenopiles (e.g., *Thalassiosira* and *Phaeodactylum* species) and alveolates (e.g., *Vitrella* and *Symbiodinum*) also encode a hemolysin III-like protein. Subcellular predictors suggest that this protein functions in the endomembrane system and contains multiple membrane-spanning domains (Additional File 1). In phylogenetic analysis, the *S. salmonicida* sequences branch together with maximum support (BV=100) with the fornicate sequences and a selection of uncultured bacteria (BV=94). Some parabasalid sequences were also identified but branch in a different part of the tree separated from other metamonads by at least two strongly supported bipartitions. Much like the SCPs, there are no closely branching sequences that have characterized representatives, making it difficult discern the function of this protein. Curiously, not all of the organisms represented here are pathogens and therefore likely do not encounter hemocytes. However, it is likely that these organisms routinely encounter other microbes in their environments that could be the target of these pore-forming toxins.

We next examined genes encoding proteins belonging to two efflux pump families: the major facilitator superfamily 1 protein (OrthoMCL: OG5_126664) and the MatE family (OG5_202696, OG5_127776, OG5_201094, OG5_202536; Na+-driven multidrug efflux pump). *S. salmonicida* encodes 12 gene copies of MFS1 proteins most of which are upregulated in OXY and NAO cells (Additional File 1). All of the *S. salmonicida* MFS1 proteins are predicted to contain membrane-spanning regions and at least four of these proteins are predicted to function at the cell membrane by BUSCA. In other organisms, these transmembrane proteins are responsible for the expulsion of drugs or other molecules [145,146] and has even been implicated in the oxygen stress response of some fungal pathogens [147]. We surveyed the *nr* database for eukaryotic and prokaryotic homologues of these proteins. While these proteins are found across the tree of bacteria and archaea, we could only detect eukaryotic homologues in fornicates (e.g., *Giardia*, *Chilomastix*, *Dysnectes*, and *Ergobibamus* species). These eukaryotic sequences branched together with strong support (BV= 100; Additional File 6) directly sister to two Asgard archaea (BV=89) sister to a clade of bacteria of mixed taxonomic affiliation (e.g., actinobacteria, proteobacteria and firmicutes). The lack of other eukaryotic homologues of these genes suggests metamonads likely acquired these genes by lateral gene transfer from a prokaryotic donor.

Finally, we investigated the MatE family of efflux pumps. MatE proteins are ubiquitously present across the tree of life and are used for the cation-mediated transport of various substrates. Some bacterial pathogens use these pumps to expel harmful drugs such as antibiotics [148]. *Spironucleus* has 13 genes that encode a MatE-like domain belonging to the NorM subfamily of MatE proteins determined using NCBI Conserved Domain database tool. Most of these genes are upregulated in OXY and NAO cells (Additional File 1). All of the genes encoding *S. salmonicida* MatE proteins are predicted to contain membrane-spanning regions and at least one of these proteins are predicted to function at the cell membrane by BUSCA. To investigate the evolutionary history of these proteins, we performed phylogenetic analysis of the closest prokaryotic and eukaryotic homologues to the *S. salmonicida* genes (Additional File 6). We detected genes encoding MatE-like proteins in free-living (e.g., *Chilomastix*, *Dysnectes* and *Carpediemonas*) and parasitic (e.g., trichomonads, *Blastocystis*, *Giardia*) lineages. Surprisingly, the eukaryotic sequences group together with strong support (BV=99) and are sister to clades composed of bacterial, and some archaeal, sequences (BV=65, BV=95). The absence of these proteins in other eukaryotes suggest that these genes might have been acquired by lateral gene transfer, from a firmicute-like bacterium.

# SUPPLEMENTARY METHODS

## *Construction of epitope-tagged* S. salmonicida

Since the carotenoid isomerase gene (SS50377_15222) does not contain introns, we used genomic DNA as a template for PCR. Briefly, the 375 bp upstream region and the entire coding sequence of SS50377_15222 was amplified using CaroIS-F (TATACGCGTGTATATTGTGTTGCTAAAGTTGCATTTTATACG) and CaroIS-R (TATGCGGCCGCTTCACTGCCTCAATTATTCTAGGGATC) using Phusion Hot Start II High-fidelity DNA polymerase (Phusion HS II) (Thermo Scientific) using the manufacturer's recommendations (98°C 3 min; 32 cycles of [98°C 15 s; 65°C 15 s; 72°C 1.10 min]; 72°C for 3 min). The resulting amplicon (2069 bp) was cloned with *Mlu*I and *Not*I restriction sites into the pSpiro-BSR-2xOLLAS expression vector [42] .The final construct was transfected into *S. salmonicida* using electroporation and transfectant cells were established and maintained by selection using 15 µg/ml blasticidin S as described [42].

## *Immunofluoresence microcopy*

The *S. salmonicida* transfectants were attached to poly-lysine coated microscope slides, fixed by 2% paraformaldehyde and prepared for immunofluorescence as described previously [42]. The cells were stained using rabbit anti-OLLAS (1:2000) (catalog no. A01658; GenScript) in blocking buffer (PBS, 0.1% Triton X-100, 3% bovine serum). The rabbit anti-OLLAS antibody was detected by goat anti-rabbit Alexa Fluor 594 (1:250) (catalog no. A-11037; Invitrogen). The slide was incubated for 1 h at room temperature, washed eight times with 15 µl wash buffer (PBS plus 0.1% Triton X-100), and mounted with 3 µl of VectaShield medium containing 4′,6-diamidino-2-phenylindole (DAPI) (catalog no. H-1200; Vector Laboratories). A coverslip was placed over the wells and sealed with nail varnish. The slides were viewed using a Zeiss Axioplan 2 fluorescence microscope. The images were processed using the software Zen 2.5 (Carl Zeiss GmbH).
