# Supplementary material for: Oxygen induces the expression of invasion and stress response genes in the anaerobic salmon parasite Spironucleus salmonicida
Source: BMC Biol. 2019 Mar 1;17:19. doi: 10.1186/s12915-019-0634-8 (PMC6397501; doi:10.1186/s12915-019-0634-8)
Supplement: Supplementary file 10 — Figure S6. Expression patterns of S. salmonicida transcription factors in OXY and NAO cells. Evolutionary gene network of all differentially expressed transcription factors in OXY (purple) and/or NAO (orange) cells based on sequence similarity. Each node represents one gene encoding a cysteine-rich protein where edges were weighted by pair-wise sequence identity. Upregulated and downregulated genes are shown in red and blue respectively. Circle nodes with or without dots represent genes that were uniquely differentially regulated in OXY or NAO cells respectively; square nodes represent genes that were similarly differentially regulated in OXY and NAO cells. A summary of the gene expression changes for each condition are shown in the bottom right corner of the network with upregulated, downregulated and non-insignificant/no change (n.s./n.c) represented as up arrowheads, down arrowheads, or dashes respectively. (PDF 805 kb) [file 12915_2019_634_MOESM10_ESM.pdf]

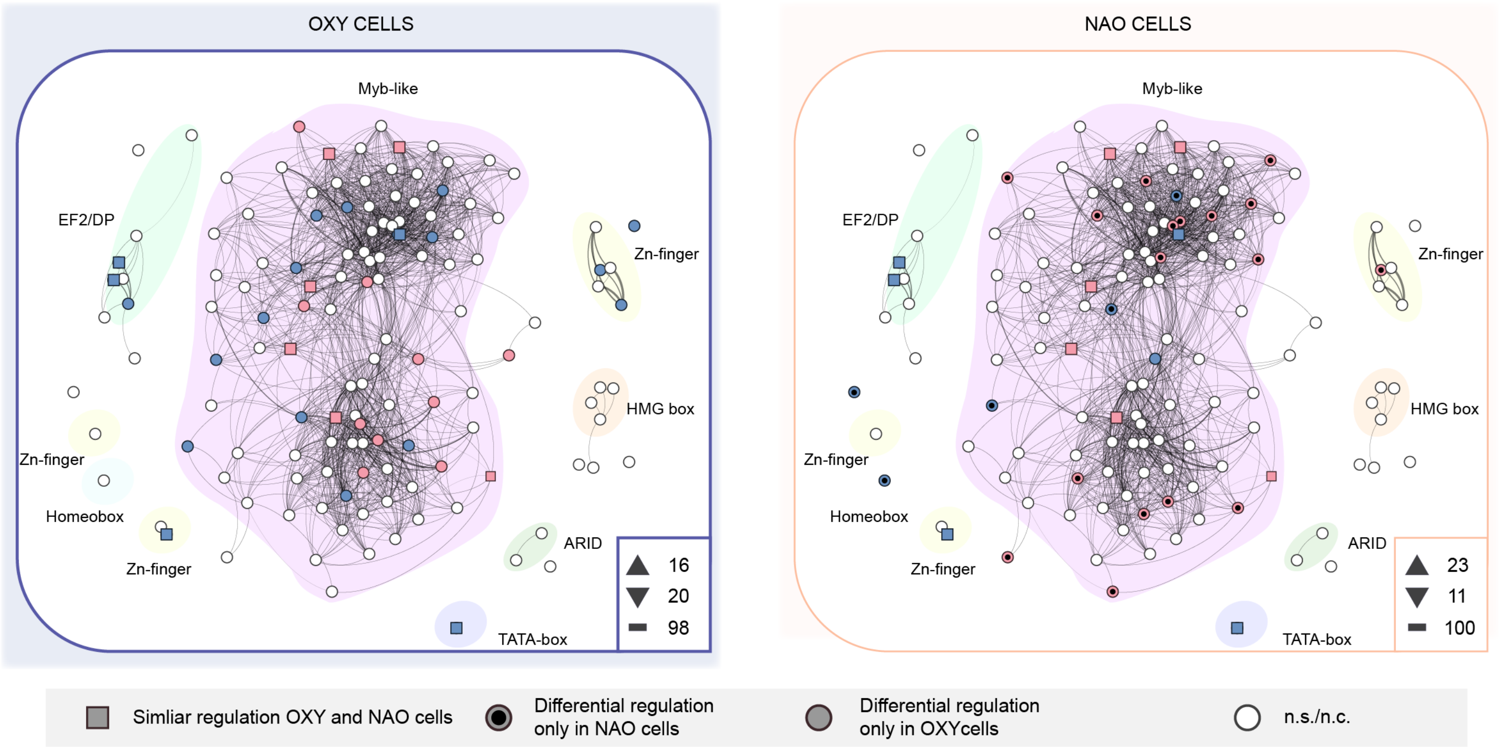

OXY CELLS
Myb-like
EF2/DP
Zn-finger
HMG box
Zn-finger
Homeobox
Zn-finger
ARID
TATA-box
16
20
98
NAO CELLS
Myb-like
EF2/DP
Zn-finger
HMG box
Zn-finger
Homeobox
Zn-finger
ARID
TATA-box
23
11
100
Simliar regulation OXY and NAO cells
Differential regulation only in NAO cells
Differential regulation only in OXYcells
n.s./n.c.
