# Supplementary material for: Oxygen induces the expression of invasion and stress response genes in the anaerobic salmon parasite Spironucleus salmonicida
Source: BMC Biol. 2019 Mar 1;17:19. doi: 10.1186/s12915-019-0634-8 (PMC6397501; doi:10.1186/s12915-019-0634-8)
Supplement: Supplementary file 12 — Differential expression workflow. R markdown file of data import and plot generation for DESeq2 analyses. (PDF 61 kb) [file 12915_2019_634_MOESM12_ESM.pdf]

Transcriptomic analysis of Spironucleus salmonicida in response to oxygen stress with DESeq2

DESeq2 workflow for transcriptome analysis performed in Stairs et al. (in preparation). RNA was isolated from Spironucleus cells grown anaerobically (Control), in the presence of oxygen for 1 hour (OXY) and in media lacking antioxidants (NAO) (n=4 each).

Analysis performed following tutorials availble on bioconductor: https://bioconductor.org/packages/release/bioc/vignettes/DESeq2/inst/doc/DESeq2.html#transcript-abundance-files-and-tximport-input

Import of necessary libraries and metadata

```
suppressMessages(library(DESeq2))
library("ggplot2")
```

Provide sample metadata file "samples.txt". Here, Control = anaerobic cells, NAO = no antioxidants and OXY = oxygen.

```
samples <- read.table("samples.txt", header = TRUE)
print(samples)
```

```
##                   run condition
## 1  SSCo01_S18.quant   Control
## 2  SSCo02_S19.quant   Control
## 3  SSCo03_S20.quant   Control
## 4  SSCo04_S21.quant   Control
## 5  SSNA01_S26.quant       NAO
## 6  SSNA02_S27.quant       NAO
## 7  SSNA03_S28.quant       NAO
## 8  SSNA04_S29.quant       NAO
## 9  SSOx01_S22.quant       Oxy
## 10 SSOx02_S23.quant       Oxy
## 11 SSOx03_S24.quant       Oxy
## 12 SSOx04_S25.quant       Oxy
```

To make sure transcript IDs and gene IDs are consisent, we provide a transcript ID to gene ID mapping file necssary for import of salmon count data to DESeq2.

```
tx2gene <- read.table("tx2gene"))
```

Import of salmon quantification files to DESeq2

Next, import the quantification files (quant.sf) from the salmon output into a varialble 'files'. Each sample is found in a subfoldfer (samples$run) in a parent directory 'dir'.

```
files <- file.path(dir, samples$run, "quant.sf")
names(files) <- paste0(samples$run)
```

Reformat the salmon quantification data in a format suitable for DESeq2.

```
txi.salmon <- tximport(files, type = "salmon", tx2gene= tx2gene)
```

Import salmon quantification data into a DESeq2 object (ddsTxi) that is re-leveled against control samples containing only samples that had non-zero read counts. Run DESeq2.

```
ddsTxi <- DESeqDataSetFromTximport(txi.salmon, colData = samples, design=~condition)
ddsTxi$condition <- relevel(ddsTxi$condition, ref="Control")
dds <- ddsTxi[which(rowSums(counts(ddsTxi))>0),]
dds<- DESeq(dds)
```

Table & Plot generation

Pair-wise comparisons with control

We performed pair-wise comparisons for the two conditions (OXY and NAO) against the controls and stored these DESeq2 objects as 'results' variables `res.1hvsC` and `res.naovsC` respectively. These objects we converted to dataframes and combined in 'combined_table_all_genes'. This dataframe was exported into a tsv file and additional analyses were performed in Microsoft Excel (Additional File 1).

```
res.1hvsC<-results(dds,contrast=c("condition", "Oxy", "Control"))
res.naovsC<-results(dds,contrast=c("condition","NAO", "Control"))
combined_table_all_genes <- cbind(as.data.frame(res.1hvsC), as.data.frame(res.naovsC))
```

Data transformation and PCA analysis

We used ‘rlog transformation’ for PCA plot visualizations. The rlog transformation of the data is stored in the `rld` variable.

```
rld <- rlog(dds, blind=TRUE)
```

Using modules from ggplot2, we generated the PCA plot shown in Figure 1B.

```
pcaData <- plotPCA(rld, intgroup=c("condition"), returnData=TRUE)
percentVar <- round(100 * attr(pcaData, "percentVar"))

PCA<- ggplot(pcaData, aes(PC1, PC2, color=condition)) +
  geom_point(size=3) +
  scale_colour_brewer(palette="Set2")+

  xlab(paste0("PC1: ",percentVar[1],"% variance")) +
  ylab(paste0("PC2: ",percentVar[2],"% variance")) +
  coord_fixed()
```

Volcano plots

For the volcano plots, the results objects (`res.1hvsC` and `res.naovsC`) were filtered to only include genes with significant q-values (less than 0.05) to generate `res.1hvsC.Sig` and `res.naovsC.Sig`. These objects were filtered again to only include genes with an absoluted fold change greater than 1.0 to generated `res.1hvC.SigFC` and `res.naovC.SigFC`.

```
res.1hvC.Sig <- res.1hvsC[ which(res.1hvsC$padj < 0.05), ]
res.1hvC.SigFC <- res.1hvC.Sig[ which(res.1hvC.Sig$log2FoldChange >1.0 | res.1hvC.Sig$log2FoldChange < -1.0)
, ]

res.naovC.Sig <- res.naovsC[ which(res.naovsC$padj < 0.05), ]
res.naovC.SigFC <- res.naovC.Sig[ which(res.naovC.Sig$log2FoldChange >1.0 | res.naovC.Sig$log2FoldChange < -
1.0), ]
```

Next, these objects were converted into dataframaes for generating volcano plots `oxy_plot` and `nao_plot`

```
res.1hvC.SigFC_df <- as.data.frame(res.1hvC.SigFC)
res.naovC.SigFC_df <- as.data.frame(res.naovC.SigFC )

oxy_plot <- ggplot(res.1hvC.SigFC_df, aes(log2FoldChange, -log10(padj))) + geom_point(size=0.5)
nao_plot <- ggplot(res.naovC.SigFC_df, aes(log2FoldChange, -log10(padj))) + geom_point(size=0.5)
```

Using in-house annotations (file=ColourMe), we coloured various genes based on the predicted function (Figure 1C). These plots were written to SVG files and colours stylized in Adobe Illustrator (not shown).

```
colour_me <- as.data.frame(read.table("ColourMe", row.names=1))
res.1hvC.SigFC_df$genename <- rownames(res.1hvC.SigFC_df)

oxy_plot_colour <- oxy_plot + geom_point(data=filter(res.1hvC.SigFC_df[rownames(colour_me),], padj<0.05), si
ze=1)
nao_plot_colour <- nao_plot + geom_point(data=filter(res.1hvC.SigFC_df[rownames(colour_me),], padj<0.05), si
ze=1)
```
